# A 3D-1D-0D multiscale model of the neuro-glial-vascular unit for synaptic and vascular dynamics in the dorsal vagal complex

**DOI:** 10.1007/s00285-025-02317-7

**Published:** 2025-12-01

**Authors:** Alexander Hermann, Tobias Köppl, Andreas Wagner, Arman Shojaei, Barbara Wohlmuth, Roland Aydin, Christian J. Cyron, Roustem Miftahof

**Affiliations:** 1 Institute of Material Systems Modeling, Helmholtz-Zentrum Hereon, Max-Planck-Str. 1, Geesthacht, 21502 Germany; 2 CAU Innovation GmbH, Fraunhoferstraße 13, 24118 Kiel, Germany; 3 Fraunhofer-Institut FOKUS, Kaiserin-Augusta-Allee 31, 10589 Berlin, Germany; 4 Department of Mathematics, Technical University of Munich, Garching, Germany; 5 Institute for Continuum and Material Mechanics, Hamburg University of Technology, Eissendorfer Strasse 42, 21073 Hamburg, Germany

**Keywords:** Multiscale modeling, Neuro-Glial-Vascular unit, Dorsal Vagal Complex, Neurovascular regulation, Synaptic dynamics, Vascular tone regulation, Primary: 92C20, Secondary: 92C35, 76Z05, 35Q92

## Abstract

Cerebral blood flow regulation is critical for brain function, and its disruption is implicated in various neurological disorders. Many existing models do not fully capture the complex, multiscale interactions among neuronal activity, astrocytic signaling, and vascular dynamics, especially in key brainstem regions. In this work, we present a 3D-1D-0D multiscale computational framework for modeling the neuro-glial-vascular unit (NGVU) in the dorsal vagal complex (DVC). Our approach integrates a quadripartite synapse model, which captures the dynamic interactions among excitatory and inhibitory neurons, astrocytes, and vascular smooth muscle cells, with a hierarchical description of vascular dynamics that couples a three-dimensional microcirculatory network with a one-dimensional macrocirculatory representation and a zero-dimensional synaptic component. By linking neuronal spiking, astrocytic calcium and gliotransmitter signaling, and vascular tone regulation, our model reproduces key features of neurovascular regulation and elucidates the feedback loops that help maintain cerebral blood flow. Simulation results demonstrate that neurotransmitter release triggers astrocytic responses that modulate vessel radius, thereby influencing local oxygen and nutrient delivery. This integrated framework provides a robust and modular platform for future investigations into the pathophysiology of cerebral blood flow regulation and its role in autonomic control, including the regulation of gastric function.

## List of Acronyms

**Table 1:** Acronyms used in the remainder of this paper

Acronym	Full name	Acronym	Full name
3D-1D	coupling of a three-dimensional and one-dimensional PDE	LHS	Latin Hypercube Sampling
ACSF	artificial cerebrospinal fluid	MAP	mean arterial pressure
AMPA	$\alpha$-amino-3-hydroxy-5-methyl-4-isoxazolepropionic acid	MLCK	myosin light-chain kinase
AP	action potential	MLCP	myosin light-chain phosphatase
APV	(2R)-amino-5-phosphonopentanoate	NADH	nicotinamide adenine dinucleotide (hydrogen)
ATP	adenosine triphosphate	NADP	nicotinamide adenine dinucleotide phosphate
BBB	blood-brain barrier	NADPH	nicotinamide adenine dinucleotide phosphate (hydrogen)
BBP	Blue Brain Project	NBC	sodium bicarbonate cotransporter
BK	large-conductance $\hbox {Ca}^{2+}$-activated $\hbox {K}^+$ channel	NGV	neuro-glia-vasculature
BRB	blood-retina barrier	NGVU	neuro-glial-vascular unit
CaM	calmodulin	NMDA	N-methyl-D-aspartate
cAMP	cyclic adenosine monophosphate	nNOS	neuronal nitric oxide synthase
CBF	cerebral blood flow	NO	nitric oxide
cGMP	cyclic guanosine monophosphate	NOS	nitric oxide synthases
CICR	Calcium-induced calcium release	NTS	nucleus tractus solitarius
$\hbox {CO}_2$	carbon dioxide	NVU	neurovascular unit
CPP	cerebral perfusion pressure	$\hbox {O}_2$	oxygen
CVR	cerebrovascular resistance	OAT	One-at-a-Time
DVC	dorsal vagal complex	PDE	partial differential equation
$\hbox {EC}_{{50}}$	half-maximal effective concentration	PICA	posterior inferior cerebellar artery
ECs	endothelial cells	$\hbox {PIP}_2$	phosphatidylinositol 4,5-bisphosphate
EDHF	endothelium-derived hyperpolarizing factor	PLC	phospholipase C
eNOS	endothelial nitric oxide synthase	$\hbox {PLC}\delta$	phospholipase C delta isoform
EPSC	excitatory post-synaptic current	PMCA	plasma membrane $\hbox {Ca}^{2+}$ ATPase
EPSP	excitatory post-synaptic potential	PRCC	Partial Rank Correlation Coefficient
ER	endoplasmic reticulum	PSP	post-synaptic potential
ET-1	endothelin-1	PUFAs	polyunsaturated fatty acids
GABA	$\gamma$-aminobutyric acid	RNA-Seq	RNA sequencing
Glu	glutamate	ROS	reactive oxygen species
ICP	intracranial pressure	SEM	scanning electron microscopy
$\hbox {IP}_3$	inositol trisphosphate	SERCA	sarco/endoplasmic reticulum $\hbox {Ca}^{2+}$-ATPase
$\hbox {IP}_3$-3K	inositol trisphosphate 3-kinase	SMCs	smooth muscle cells
$\hbox {IP}_3$-5P	inositol trisphosphate 5-phosphatase	SR	sarcoplasmic reticulum
IPSC	inhibitory post-synaptic current	TCA	tricarboxylic acid
IPSP	inhibitory post-synaptic potential	TRP	transient receptor potential
KCC1	potassium chloride cotransporter	VOCC	voltage-operated calcium channels
KIR	inward-rectifying potassium channels	WSS	wall shear stress
L-Arg	L-arginine		

## Introduction

Understanding perception, cognition, learning, memory and consciousness in the brain are the prime objectives of neuroscience. Until now, research on the brain has been dominated by both experimental and theoretical reductionism, which emphasizes detailed knowledge of its structure and function at a high-resolution level. Successful examples of this approach include a computationally intensive reconstruction of morphological constituents in a cubic millimeter of the human temporal cortex at a nanometer scale (Shapson-Coe et al. 2024); the uncovering of cellular diversity at the transcriptomics level (Schaeffer and Iadecola 2021); real-time monitoring of the dynamics of synaptic plasticity using genetically encoded, intensiometric fluorescence indicators (Son et al. 2024). Although purely reductive strategies provide amazing insights into the functional intricacies of biological matter, they unequivocally neglect the context in which these processes operate. This severely limits the ability of reductionism to explain how and why the brain behaves as it does. In parallel with reductionist advances, large-scale open ecosystems now support integrative, multiscale unit (NGVU) investigations; notably, the Blue Brain Project (BBP) toolchain includes NEURON for morphologically detailed neuronal biophysics and STEPS for stochastic reaction–diffusion at subcellular and synaptic scales, with related efforts for astrovascular/cerebrovascular workflows Awile et al. (2022); Chen and De Schutter (2017). Complementing these simulators, community resources target neuro-glia-vasculature (NGV) structural assembly and reconstruction, including the ArchNGV repository and a digital NGV reconstruction of cortical tissue Zisis et al. (2021). In addition, the Blue Brain Neuro-Glia-Vasculature (NGV) Model Repository and the NGV Portal curate implementations, datasets, and documentation for NGV/NGVU modeling [7,8]. Even so, the reductive trend continues to prevail in *in vitro* and *in vivo* studies of brain function under physiological and disease states. It is attractive from experimental and theoretical perspectives to investigate a single target in isolation, such as dopamine neurotransmission in the pathogenesis of Parkinson’s disease, neuronal activity in the pathophysiology of epilepsy, or cerebral blood flow (CBF) in stroke and vascular dementia patients. However, being deterministic in nature, the underlying physiological processes run at multi-hierarchical levels and cannot be accounted for by reductionism. This shift has led to a change in the paradigm of studying the finely parcellated brain, moving from a reductionist approach to an integrative analysis at different levels of its organization.

The concept of the neurovascular unit (NVU) as a functional element of the brain was proposed and formalized in 2001 at the inaugural Stroke Progress Review Group meeting of the National Institute of Neurological Disorders and Stroke (Iadecola 2017). Initially, it was defined morphologically as a hetero-cellular structure composed of neurons, endothelial cells (ECs), vascular smooth muscle cells (SMCs), and the extracellular matrix. The NVU was assigned *a priori* properties of functional homogeneity and generality throughout the brain (Schaeffer and Iadecola 2021). Over the years, the concept has evolved from its “canonical” form to a NGVU, incorporating additional cellular constituents such as astrocytes and pericytes, to highlight the multi-functional, -dimensional, and -cellular coordinated function of the brain. Recent reviews synthesize glial–vascular unit biology and its alterations across central nervous system disorders, providing broader context for NGVU modeling Yao et al. (2023). Complementing that broader synthesis, Kugler et al. frame a glia-centric “neuro–glial–vascular unit,” highlighting astrocyte–endothelium–pericyte crosstalk, blood–brain and blood–retina barrier (BBB/BRB) roles, and NVU development and dysfunction across brain and retina (Kugler et al. 2021). In addition, Ding et al. review microglia within the NVU and summarize *in vitro* NVU platforms (microglia–neuron co-cultures and organotypic preparations) that recapitulate morphological features and microglia–neuron signaling (Ding et al. 2021).

Recent single-cell RNA sequencing (RNA-Seq) analyses have proven the molecular heterogeneity of brain cells (Tripathy et al. 2017). This has inferred the functional diversity of NGVUs that depends on topological cell arrangements, regional specialization, and the metabolic demands of different brain areas. The most investigated function of the NVU is neurovascular coupling, i.e., the matching of blood flow to neuronal metabolic demands, also known as functional hyperemia. Although the large vessel architecture of the brain is established at birth, the intracerebral vascular network grows postnatally, known as capillary angiogenesis, to match cortical expansion (Marín-Padilla 2012). Two main cell types, ECs and pericytes, embedded in the basal lamina, constitute the wall of capillaries. ECs linked via gap junctions create a smooth luminal syncytium of the capillaries. These are ensheathed externally by pericytes. These cells have variable morphology ranging from circumferential, longitudinal to amoeboid depending on the location along the vascular tree (Hartmann et al. 2015). Pericytes mediate precise local changes of capillary diameter in response to excitatory/inhibitory signals arriving from the neurons and astrocytes. Coupled via gap junctions, pericytes and ECs together sustain the conduction of locally induced signals along the capillary and arteriolar networks and thus regulate CBF over space and time (Hall et al. 2014; Longden et al. 2017; Cai et al. 2018; Rungta et al. 2018; Emerson and Segal 2000a, b; Otani et al. 2023; Ii et al. 2020).

During capillary angiogenesis, astrocytes undergo massive proliferation. The basal lamina of blood vessels and the astrocyte-secreted basal lamina merge to form a common cerebrovascular basal lamina. This sets close contacts between the ECs, pericytes, and astrocytes. Mature astrocytes vary in density by brain region and display regional heterogeneity in morphology (Emsley and Macklis 2006; Bayraktar et al. 2020). Interconnected via gap junctions, they form a topologically distinct network that ensheathes through astrocytic perivascular endfeet processes, 99.7% of the capillary network and, concurrently, through its peri-synaptic processes, enwraps pre- and post-synaptic terminals of neurons, forming *tripartite synapses* (Bushong et al. 2002; Kofuji and Newman 2004; Tsai et al. 2012; Mathiisen et al. 2010). These provide efficient connections between the neuronal, astrocytic, and vascular networks and drive neurovascular coupling within the NGVU (Mishra 2017; Hautefort et al. 2019; Presa et al. 2020). Astrocytes, ECs, and pericytes sense signaling within NGVUs and adjust CBF according to metabolic demands. The release of glutamate (Glu) and adenosine triphosphate (ATP) at the tripartite synapse triggers an influx of extracellular $\hbox {Ca}^{2+}$ to astrocytes. The increase in cytosolic $\hbox {Ca}^{2+}$ activates the release of gliatransmitters. Their biological effects depend on the site of action, the types of activated receptors, and the ligand concentration. Thus, nitric oxide (NO) and epoxyeicosatrienoic acid have been shown to produce strong capillary vasodilation. 20-hydroxyeicosatetraenoic acid, prostaglandin E2, and $\hbox {Ca}^{2+}$ ions have potent vasoconstrictive (Biesecker et al. 2016; Magaki et al. 2018; Thakore et al. 2021), while astrocyte-derived ATP and its metabolite adenosine exhibit bidirectional effects on the capillary wall (Zaritsky et al. 2000; Filosa et al. 2006; Longden and Nelson 2015). Intriguingly, astrocytes can also perceive changes in capillary blood pressure and, in turn, amend neuronal firing activity (Kim et al. 2015, 2016). This vasculo-neuronal hypothesis may present a mechanism through which body state can dictate, or at least modulate, brain function (Moore and Cao 2008; Filosa et al. 2016; Presa et al. 2020). ECs react to changes in intracapillary pressure/flow and wall shear stress by releasing NO, endothelial factor-1, prostacyclin, and proteases. These cause pericyte relaxation, break up the extracellular matrix, induce vascular remodeling, and neovascularization that alter CBF to meet urgent requirements.

The study of nonlinearity, sensitivity to initial and state variables, stochasticity, and chaotic behavior of the NGVU is central to understanding and identifying the key determinants of the (patho)physiology of the brain. Mathematical modelling of NGVUs has witnessed impressive advances within the last decade due to an explosion in the amount and quality of experimentally available data. Two broad classes of models have emerged, i.e., i) neuron abstract, and ii) neuron morphologically and physiologically extended network models. While the first class captures essential architectural and electrical neuronal features, the second class accentuates the internal physiological signal processing underlining the repertoire of electrical activity observed *in vivo*. The number of publications on the subject is continuously increasing and it is beyond our scope to cite and evaluate critically each model and their produced results. We rather consider it the reader’s responsibility to stay updated on progress in the field while exercising thorough impartial scientific judgment regarding the veracity, biological plausibility, and significance of the models. From our literature review, we conclude that at the time of writing there are no models of the NGVU that capture its role as a fundamental morpho-functional unit of the human brain. Current models, without exception, are based on fragmentary analyses of constitutive components, and not a holistic representation of the unit with its complex biology. In parallel, open community toolchains furnish robust components for cellular and subcellular simulation–NEURON for morphologically detailed electrophysiology and STEPS for stochastic reaction–diffusion–yet these primarily address components rather than NGVU-level integration; the present work instead articulates and evaluates NGVU-specific cross-scale couplings (astrocyte–synapse–endothelium/pericyte) and phenomenology-grounded parameterization, while remaining solver-agnostic Awile et al. (2022); Chen and De Schutter (2017). Consequently, these models do not support investigations or provide answers to questions about the brain’s perception and cognition in health and disease (El-Bouri et al. 2021; Chen et al. 2024). To address this gap, we introduce a 3D-1D-0D multiscale computational tool designed to simulate the dynamics of the NGVU in a more integrated manner. Our model is built upon established biological principles and existing research, with validation currently focused on qualitative alignment with these foundations. However, due to the complexity of the system and the limitations of available experimental data, full quantitative validation remains a future direction to enhance the model’s predictive accuracy.

**Fig. 1:**
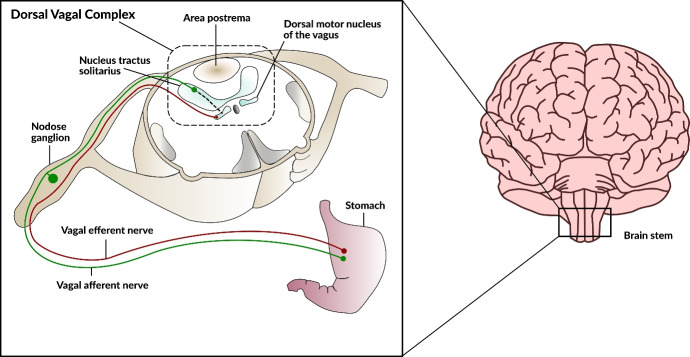
Schematic representation of the dorsal vagal complex. A detailed view of the dorsal vagal complex is depicted, including the stomach, vagal afferent and efferent nerves, nodose ganglion, nucleus tractus solitarius, area postrema, and the dorsal motor nucleus of the vagus (adapted from (Zaved Waise et al. 2018))

Within this study, we direct our attention to the dorsal vagal complex (DVC), a key brainstem structure located in the medulla oblongata. The DVC plays a pivotal role in autonomic control by integrating visceral sensory and motor signals that regulate parasympathetic outflow to the cardiovascular, respiratory, and gastrointestinal systems (Mahdi et al. 2013). It works closely with neighboring nuclei, including the nucleus tractus solitarius (NTS), to coordinate reflexes such as baroreception (blood pressure regulation) and gut motility. Because the DVC must respond rapidly to changing homeostatic demands, studying its local neuro-glial-vascular interactions is especially relevant (De Pittà et al. 2011). Fluctuations in autonomic activity require fine-tuned mechanisms to ensure adequate blood flow and metabolic support, making the DVC an ideal site for investigating the core principles of multicellular coordination in the brainstem (Coggan et al. 2018; Choi and Mihalas 2019). To facilitate a clearer understanding of our multi-scale modeling framework, Figure 1 provides a schematic illustration that depicts the stepwise construction of the model–from the macrovascular network (including the carotid, vertebral arteries and the circle of Willis), through the mesoscale arterial tree, to the microvascular unit representing a capillary vessel in the DVC, and finally the integration of the quadripartite synapse model with dynamic vessel radius regulation. By focusing on this region, we also hope to shed light on how disruptions in these processes could contribute to disorders affecting autonomic functions. This DVC-focused scope complements portal-curated NGV exemplars and workflows–largely cortical in emphasis–made available through the Blue Brain NGV Model Repository and the NGV Portal (Bambrick et al. 1997; Barnes and Charkoudian 2021).

**Fig. 2:**
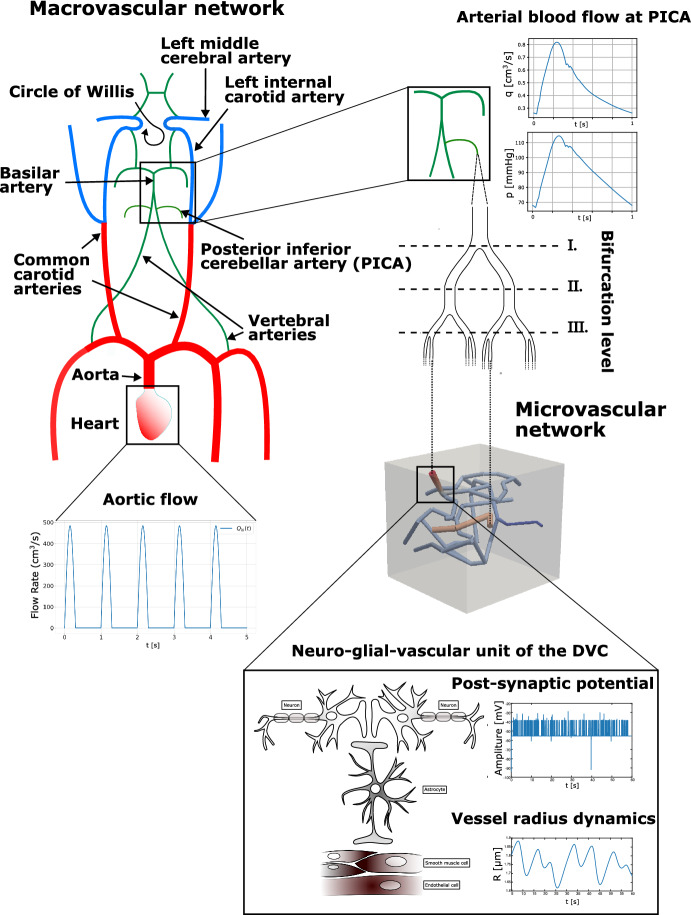
Graphical Abstract. Schematic overview of our novel multiscale modeling framework for the NGVU in the DVC. The top panel shows the heart generating a pulsatile blood flow at one beat per second that is transmitted through major arteries including the common carotid, vertebral, and internal carotid arteries and collected in the circle of Willis. The arterial blood then enters the posterior inferior cerebellar artery (PICA) to supply the DVC via a series of symmetric bifurcations in the mesoscale arterial tree. The bottom panel shows a detailed view of the local neuro-glial-vascular unit where the quadripartite synapse model captures interactions among excitatory and inhibitory neurons, astrocytes, and vascular smooth muscle cells. This integration dynamically regulates vessel radius and optimizes oxygen delivery to surrounding tissue through a one-dimensional to three-dimensional coupling approach

The aim of this paper is to provide a modular framework for constructing a comprehensive *in silico* model of the NGVU, which represents the first integrated model of its kind for the DVC. We begin with a gliovascular module in which astrocytic glia supports the function of associated cerebrovascular segments. A brief background of the major biological components that justify the working assumptions underlying our mathematical modelling of the essential physiological processes is presented in Section 2. This is followed by a description of a spatiotemporal computational model of the NGVU in Section 3, which includes discussions of the blood flow model, the quadripartite synapse model, the components of the neurovascular coupling model, and their integration. Numerical simulation results are presented in Sect. 4. Section 5 discusses the findings and Section 6 summarizes the contributions and suggests directions for future research. For a concise visual summary of our approach and its novelty, see the Graphical Abstract in Fig. 2.

## Biological foundations of Neuro-Glial-Vascular interactions

### Anatomical and morphological components of the NGVU

The NGVU consists of structurally and functionally interconnected components essential for modeling brain physiology. Key elements include neurons, glial cells (especially astrocytes), SMCs, ECs, and blood vessels. These components work in concert to regulate cerebral blood flow, vascular tone, and neural activity, making their anatomical, morphological, and physiological characteristics vital for constructing a biologically accurate mathematical model.

Neurons, the fundamental morphological and functional units of the brain, number approximately $10^{11}$ in the adult human brain. Structurally, they comprise the soma, axon(s), dendrites, nerve terminals, and spine buds (Heinrich et al. 2014). The soma, measuring ${3}\,\upmu \hbox {m}$ to ${18}\,\upmu \hbox {m}$ in diameter (Chudler 2013), contains essential organelles such as the nucleus, endoplasmic reticulum (ER), mitochondria, and ribosomes, which sustain neuronal functionality (Zilles and Tillmann 2011; Ariyo and Akter 2024). Axons, cylindrical extensions of the soma, have diameters ranging from ${0.1}\,\upmu \hbox {m}$ to over ${10}\,\upmu \hbox {m}$ and lengths of ${20}\,\upmu \hbox {m}$ to ${200}\,\upmu \hbox {m}$, with collateral branches exceeding ${10}\,\upmu \hbox {m}$ (Lee et al. 2019; Samuels et al. 2003). They end in nerve terminals or terminal boutons, about 1–${2}\,\upmu \hbox {m}$ in diameter (Zilles and Tillmann 2011). Axons can be myelinated or unmyelinated, depending on the presence of a myelin sheath (Zilles and Tillmann 2011; Samuels et al. 2003). Dendrites (from Greek $\delta \acute{\epsilon } \nu \delta \rho o \nu$, meaning *tree*) are shorter, thinner processes branching from the soma, characterized by spine buds (0.5–${2}\,\upmu \hbox {m}$ in diameter) connected by a spine neck (0.04–${1}\,\upmu \hbox {m}$ long) (Harris and Kater 1994). Unlike axons, dendrites have a poorly developed cytoskeleton and fewer neurofilaments but are rich in microtubules and microfilaments. The nerve terminals and spine buds are key sites for synaptic connections (from Greek $\sigma \acute{\upsilon } \nu \alpha \psi \iota \varsigma$, meaning *conjunction*) (Reichardt and Kelly 1983), consisting of a presynaptic terminal, a synaptic cleft (20–${30}\,\hbox {nm}$), and a postsynaptic terminal. These connections form between neurons, astrocytes, or combinations thereof via dendritic, axonal, axo-dendritic, and axo- or dendro-somatic synapses (Zilles and Tillmann 2011).

Astrocytes, oligodendrocytes, microglia, and radial cells belong to the class of glial cells (Heinrich et al. 2014; Zilles and Tillmann 2011). Astrocytes are star-shaped cells with a relatively small soma measuring 10–${20}\,\upmu \hbox {m}$ (Zilles and Tillmann 2011). Six or more major branches extend from the soma, further dividing into branchlets and leaflets, with cross-sectional diameters ranging from 10 to ${100}\,\hbox {nm}$ (Chai et al. 2017; Baldwin et al. 2024). Morphologically, astrocytes are categorized into two types: fibrous, found in white matter, and protoplasmic, located in gray matter. Both types feature fewer organelles and unbranched processes up to ${300}\,\upmu \hbox {m}$ in length (Ariyo and Akter 2024; Goenaga et al. 2023). The process endings, known as endfeet, form astro-dendritic, astro-axonal, and neuron-smooth muscle synapses within the NGVU (Kacem et al. 1998; Garman 2011; Simard et al. 2003). Astrocytes possess a well-developed ER, which serves as the primary intracellular $\hbox {Ca}^{2+}$ store, along with numerous mitochondria and an extensive microfilament network (Shigetomi et al. 2016; Sherwood et al. 2021). *In vitro* NVU models, including microglia–neuron co-cultures and organotypic slice systems, capture key aspects of NVU morphology and microglial surveillance observed *in vivo* (Ding et al. 2021).

Smooth muscle cells form the walls of cerebral blood vessels (Zilles and Tillmann 2011). These fusiform-shaped cells measure approximately ${20}\,\upmu \hbox {m}$ in length and ${6}\,\upmu \hbox {m}$ in width, playing a crucial role in regulating vascular tone and providing mechanical elasticity to the vascular wall (Hill-Eubanks et al. 2014; Wight 2008). SMCs form a syncytium through multiple gap junctions and basal laminae invaginations (Zilles and Tillmann 2011). Their contractile apparatus comprises contractile proteins, regulatory proteins, and a cytoskeleton. The primary contractile proteins are actin and myosin, organized into thin and thick filaments (myofilaments). Thin filaments, approximately ${7}\,\hbox {nm}$ in diameter, consist mainly of actin, while thick filaments, measuring 12–$15\hbox {nm}$ in diameter, are predominantly composed of myosin. Regulatory proteins such as myosin lightchain kinase (MLCK), myosin light-chain phosphatase (MLCP), calmodulin (CaM), and caldesmon, along with the intracellular arrangement of myofilaments, constitute the functional contractile apparatus (Zilles and Tillmann 2011; Chen et al. 2020).

The luminal surface of blood vessels is lined with a monolayer of ECs, forming a selective semi-permeable membrane that regulates the movement of small molecules ($\hbox {O}_2$, $\hbox {CO}_2$, $\hbox {H}_2\hbox {O}$) and ions ($\hbox {Ca}^{2+}$, $\hbox {K}^+$) between the blood and brain parenchyma. These cells also modulate cerebral blood flow, vascular tone, inflammation, thrombosis, and adhesion. Endothelial dysfunction is a key factor in the pathogenesis of cerebral small vessel disease (Zilles and Tillmann 2011; Sweeney et al. 2018). ECs synthesize and release vasoactive mediators, including NO, endothelium-derived hyperpolarizing factor (EDHF), eicosanoids, and endothelin- 1 (ET-1), to regulate vascular tone. NO and EDHF act as potent vasodilators by influencing the contractile apparatus and membrane hyperpolarization of SMCs, while eicosanoids and ET-1 function as vasoconstrictors, inducing intense smooth muscle contractions (Furchgott and Zawadzki 1980; Longden et al. 2017).

Calcium ions act as crucial secondary messengers in regulating endothelial cell processes. Under physiological conditions, ECs maintain a low cytosolic $\hbox {Ca}^{2+}$ concentration ($100\,\hbox {nmol}\,\hbox {L}^{-1}$) compared to the extracellular concentration of 1.5–$2\,\hbox {mmol}\,L^{-1}$ in the brain. Cytoplasmic $\hbox {Ca}^{2+}$ is essential for the synthesis and release of endothelium-derived factors, including NO. Agonist-driven NO release, such as by acetylcholine, is mediated by intracellular $\hbox {Ca}^{2+}$ oscillations. Mechanical stimuli sensed by mechanosensitive ion channels elevate intracellular $\hbox {Ca}^{2+}$ levels, leading to endothelium-dependent smooth muscle hyperpolarization and relaxation. Additionally, reactive oxygen species produced by ECs can activate transient receptor potential (TRP) channels, triggering $\hbox {Ca}^{2+}$ influx and dilating cerebral arteries (Tran et al. 2000; Ando and Yamamoto 2013).

### Transmitter, ion channels and electrical signal transduction

The NGVU relies on various electrochemical processes to facilitate neural communication and vascular regulation. These processes are driven by the coordinated action of neurotransmitters, ion channels, and second messengers, which underlie the generation and propagation of signals across neurons, astrocytes, and vascular components. Understanding the dynamics of these signaling mechanisms is essential for modeling the complex interactions within the NGVU, as they regulate neural activity, synaptic transmission, and vascular tone in both physiological and pathological conditions.

Transmitters are essential for the electrochemical processing of information at chemical synapses in the NGVU. Glu, the primary excitatory neurotransmitter, is used by nearly 90% of neurons and accounts for 80–90% of brain synapses. Synthesized *de novo* from glucose or via glutamine recycling, Glu is stored in vesicles and released by exocytosis (Heinrich et al. 2014). In the synaptic cleft, Glu binds to ionotropic receptors (N-methyl-Daspartate (NMDA), $\alpha$-amino-3-hydroxy-5-methyl-4-isoxazolepropionic acid (AMPA) and kainate) to alter ion channel permeability or to metabotropic receptors, which regulate intracellular signaling via G-proteins (Sahlender et al. 2014; Montana 2006). $\gamma$-aminobutyric acid (GABA), the primary inhibitory neurotransmitter, is synthesized via the GABA-shunt and operates through ionotropic GABAa and metabotropic GABAb receptors. GABAa activation causes $\hbox {Cl}^-$ influx and hyperpolarization, while GABAb reduces $\hbox {Ca}^{2+}$ influx and increases $\hbox {K}^+$ efflux, inhibiting cell excitability (Heinrich et al. 2014; Kubota et al. 1994). NO, a gaseous transmitter synthesized on demand by NO synthases, diffuses freely due to its solubility in fat and water (Alderton et al. 2001; Förstermann et al. 1998). Despite its short half-life (${2}\,\hbox {ms}$ intravascularly and up to $2\,\hbox {s}$ extravascularly), NO mediates significant physiological effects, including smooth muscle relaxation and vasodilation (Dormanns et al. 2016; Garthwaite and Boulton 1995). Endothelial cell-derived NO primarily drives vasorelaxation during rest, while neuronal NO dominates during activation, influencing arteriolar diameter by diffusing into SMCs (Dormanns et al. 2016). NO release, triggered by wall shear stress, delays the return of arteriolar radius to baseline (Thomas et al. 2001). Together, Glu and NO and mediate excitatory synaptic signaling and vascular regulation, while the interplay with inhibitory GABA ensures neural and vascular homeostasis in the NGVU (Bezzi and Volterra 2001; Heinrich et al. 2014; Dormanns et al. 2016).

In addition to signaling molecules, second messengers play a crucial role in intracellular signal transmission by forming after the binding of signaling molecules to membrane-bound or cytosolic receptors (Heinrich et al. 2014). Inositol trisphosphate $\hbox {IP}_3$, a cyclic compound derived from membrane phospholipids during the $\hbox {IP}_3$ pathway, triggers $\hbox {Ca}^{2+}$ release from the ER (Heinrich et al. 2014). ATP, apart from serving as an energy source, acts as a second messenger by binding to purinergic receptors and is co-localized with other neurotransmitters (Heinrich et al. 2014). In astrocytes, ATP facilitates $\hbox {Ca}^{2+}$ wave propagation and is released in a $\hbox {Ca}^{2+}$-dependent manner or through lysosome exocytosis, hemichannels, and anion channels (Xiong et al. 2018; Lee et al. 2015; Kang et al. 2008). Cellular membranes, composed of amphiphilic lipid bilayers with hydrophilic head groups facing outward and hydrophobic tails inward, serve as structural barriers enabling the exchange of substances between cells and their environment (Heinrich et al. 2014; Brandes et al. 2019). Ion channels, integral membrane proteins, facilitate the passage of ions like $\hbox {Na}^+$, $\hbox {K}^+$, $\hbox {Ca}^{2+}$, and $\hbox {Cl}^-$ across the lipid bilayer through selective water-filled pathways, achieving transport rates of $\hbox {10}^7$–$\hbox {10}^8$ ions per second and forming the basis of cellular excitation (Brandes et al. 2019).

The movement of ions is driven by two primary forces: the concentration gradient and the potential difference. Passive transport occurs along an electrochemical gradient, with the ion current determined by the channel’s conductivity and open probability. Greater membrane potential amplitude increases the driving force and current amplitude. Depending on channel type, ion concentration, and temperature, ion channel conductivities range from 1 to $10^{-10}$ S, while specific membrane resistances vary from $10^{12}$ to $10^9$ $\Omega$ (Brandes et al. 2019). Neurons of the NGVU model express voltage-dependent $\hbox {Na}^+$ channels, L-type and N-type $\hbox {Ca}^{2+}$ channels, $\hbox {Cl}^{-}$ channels, and large conductance $\hbox {Ca}^{2+}$-dependent $\hbox {K}^+$ channels (BK) (Ghatta et al. 2006). Calcium-induced calcium release (CICR) facilitates $\hbox {Ca}^{2+}$ release from intracellular stores (Bootman et al. 2002), while voltage-operated calcium channels (VOCC) mediate calcium entry during depolarization, crucial for neurotransmitter release and muscle contraction (Sher et al. 1991). Other critical ion transporters include nward-rectifying potassium channels (KIR), which regulate the resting membrane potential (Hibino et al. 2010), the sodium bicarbonate cotransporter (NBC) for pH and ion homeostasis (Bernardo et al. 2006), and the potassium chloride cotransporter (KCC1) for cell volume regulation (Russell 2000). Neuronal communication relies on electrical potentials, with dendrites transmitting signals to the soma, where an action potential (AP) are generated (Heinrich et al. 2014; Brandes et al. 2019). Action potentials involve sequential phases: overcoming the threshold potential, rapid depolarization (upstroke and overshoot), repolarization, and post-hyperpolarization, mediated by $\hbox {Na}_v$-channels for depolarization and $\hbox {K}_v$-channels for repolarization (Brandes et al. 2019).

Axons and dendrites function as biological cables composed of capacitor-resistor elements, with distinct roles in neural signal processing. In dendrites, the membrane time constant and length constant govern the integration and conduction of synaptic potentials to the soma, while axons employ active mechanisms to propagate the AP (Brandes et al. 2019). Unmyelinated axons typically conduct the AP at approximately 1 m/s, with conduction speed increasing as axonal diameter grows, reducing internal resistance. Human unmyelinated nerve fibers generally have a diameter of around 1 $\mu \hbox {m}$, allowing continuous excitation propagation along the axon to the synapse (Brandes et al. 2019). At chemical synapses, electrical signals are converted into chemical reactions mediated by neuro- and gliotransmitters (Heinrich et al. 2014). Synaptic transmission involves a sequence of events: nerve terminal depolarization, $\hbox {Ca}^{2+}$ influx via VOCC, diffusion of $\hbox {Ca}^{2+}$ to vesicular active sites, neurotransmitter release into the synaptic cleft, receptor binding on the postsynaptic membrane, activation of ion channels or second messenger systems, and either depolarization or hyperpolarization of the postsynaptic membrane (Brandes et al. 2019).

### Physiology of vasoconstriction, autoregulation and bioenergetics

Understanding the physiology of vasoconstriction, autoregulation, and bioenergetics is essential for accurately modeling the dynamic interactions within the NGVU. These processes govern the regulation of cerebral blood flow, vascular tone, and intracellular energy metabolism, forming the basis for predictive models of brain function. By incorporating these mechanisms, models can capture the physiological responses to neural activity, systemic changes, and metabolic demands, enabling deeper insights into neurovascular coupling and brain homeostasis.

SMCs in the blood vessel wall form a syncytium and rely on post-translational modifications for contraction due to the absence of intrinsic myosin ATPase activity. Contraction is regulated by MLCK, a $\hbox {Ca}^{2+}$/CaM-dependent kinase activated by free $\hbox {Ca}^{2+}$, which phosphorylates myosin, enabling actin-myosin interaction and force generation (Ito and Hartshorne 1990; Dormanns et al. 2015; Zilles and Tillmann 2011). Caldesmon, bound to actin-tropomyosin, is displaced upon $\hbox {Ca}^{2+}$-CaM binding, facilitating this interaction. MLCP counteracts MLCK activity, its regulation mediated by Rho-kinase inhibition or stimulation via NO through cyclic guanosine monophosphate (cGMP) and cyclic adenosine monophosphate (cAMP) production (Pappano 2013). SMCs integrate inputs from neurons, astrocytes, and ECs, primarily via $\hbox {Ca}^{2+}$ transport and voltage coupling. Their contraction or relaxation modulates vessel radius, driving vasoconstriction or dilation and playing a critical role in neurovascular regulation within the NGVU (Dormanns et al. 2015; Pappano 2013).

The cardiovascular system, consisting of the heart and blood vessels, facilitates the transport and exchange of oxygen ($\hbox {O}_2$), carbon dioxide $\hbox {CO}_2$, nutrients, electrolytes, and hormones (Zilles and Tillmann 2011). Arteries carry blood away from the heart, while veins return it, with arterioles, capillaries, and venules connecting these pathways. The aorta branches into progressively smaller arteries, forming a vascular tree where vessel diameters decrease while cross-sectional area increases. Vessel walls have three layers: the tunica intima, lined with ECs for adhesion and coagulation; the tunica media, containing SMCs and elastic fibers to regulate diameter; and the tunica externa, made of connective tissue (Zilles and Tillmann 2011). Arteries can measure up to ${3}\,\hbox {cm}$, arterioles are under ${30}\,\upmu \hbox {m}$, and capillaries are 5–${8}\,\upmu \hbox {m}$ wide, optimizing exchange due to their large cross-sectional area and a flow rate of ${0.5}\,\hbox {mm}\,\hbox {s}^{-1}$ (Zilles and Tillmann 2011; Xue et al. 2022). Four major arteries, including the internal carotid and vertebral arteries, supply the brain. Capillaries, spanning ${644}\,\hbox {km}$ with a surface area of $20\,\hbox {m}^{2}$, consist of ECs, a basal lamina, and pericytes. Smooth muscle layers in larger arteries reduce to a single layer in capillaries, where pericytes replace SMCs, separated from astrocytes by the perivascular space (Schaeffer and Iadecola 2021; Begley and Brightman 2003; Zlokovic 2008).

Autoregulation maintains nearly constant CBF within a range of $50\,\hbox {mmHg} to {160}\,\hbox {mmHg}$ by adjusting vascular tone to ensure oxygen and nutrient delivery (Elting et al. 2020; Duchemin et al. 2012; Iadecola 1993). CBF is driven by cerebral perfusion pressure (CPP), defined as the difference between mean arterial pressure (MAP) and intracranial pressure (ICP), and cerebrovascular resistance (CVR) (Presa et al. 2020). CPP typically ranges from ${60}\,\hbox {mmHg}\,{80}\,\hbox {mmHg}$, while normal ICP is $5\hbox {mmHg}\,10\,\hbox {mmHg}$ and has less impact on CPP than MAP (Presa et al. 2020). MAP, averaging blood pressure over one cardiac cycle, normally ranges from ${70}\,\hbox {mmHg}\,{100}\,\hbox {mmHg}$ but fluctuates with activity, stress, or other systemic factors (Mount and Das 2023). Autoregulation alters vessel diameter through vasodilation (increased diameter) or vasoconstriction (decreased diameter) (Peppiatt et al. 2006; Phillips et al. 2016). However, its range is limited to $\pm {20}\,\hbox {mmHg}$ from baseline with a latency of ${60}\,\hbox {s}$ (Claassen et al. 2021). Sudden or extreme arterial pressure changes induce passive CBF variations. Elevated arterial pressure increases intracellular $\hbox {Ca}^{2+}$ in SMCs, activating MLCK and triggering actin-myosin crosslinking, resulting in smooth muscle contraction and vasoconstriction (Schaeffer and Iadecola 2021; Phillips et al. 2016). Systemic factors such as posture, glucose levels, hormones, hematocrit, blood viscosity, and cold stress also influence CBF, engaging the cerebrovascular tree with segmental specificity (Claassen et al. 2021; Barnes and Charkoudian 2021).

Intracellular nutrient metabolism is fundamental for brain function, driven by biochemical pathways that depend on the delivery of glucose and oxygen, regulated by neuro-glial activity. Within cells, mitochondria play a central role in energy production through enzymatic reactions, essential for eukaryotic cells such as neurons, astrocytes, ECs, and SMCs (Cortassa et al. 2019). Metabolic pathways include glycolysis, glycogenolysis, the pentose phosphate and polyol pathways, $\beta$-oxidation, the tricarboxylic acid (TCA) cycle, ATP synthesis, ionic and redox-energy transducing processes, and reactive oxygen species (ROS) generation and scavenging (Cortassa et al. 2019). ATP is synthesized via glucose catabolism through glycolysis, the TCA cycle, and oxidative phosphorylation, where nicotinamide adenine dinucleotide (hydrogen) (NADH)-driven proton gradients power ATP generation (Senior et al. 2002). Glycolysis produces ATP both anaerobically and aerobically, generating pyruvate for the TCA cycle and intermediates for anabolic pathways (Chandel 2021). Glycogenolysis breaks glycogen into glucose-1-phosphate and glucose, providing a key glycogen utilization pathway (Panja et al. 2013). The pentose phosphate and polyol pathways support biosynthetic metabolism and redox homeostasis (Stincone et al. 2015). The ATP cycle, involving eight enzymes, links pyruvate and malate oxidation to $\hbox {CO}_2$ production and NADH generation, sustaining mitochondrial respiration and metabolic functions (Fernie et al. 2004; Fernie and Martinoia 2009). $\beta$-oxidation of polyunsaturated fatty acids (PUFAs) balances energy demand, competing with glucose as a primary oxidative substrate (Ghisla 2004). Ionic and redox-energy transducing processes couple biochemical systems through electronic energy transitions (Losada et al. 1983). ROS generation and scavenging involve oxygen radicals and oxidizing agents, influencing metabolism, mitochondrial electron transport, signal transduction, and gene expression (Bayir 2005).

**Fig. 3:**
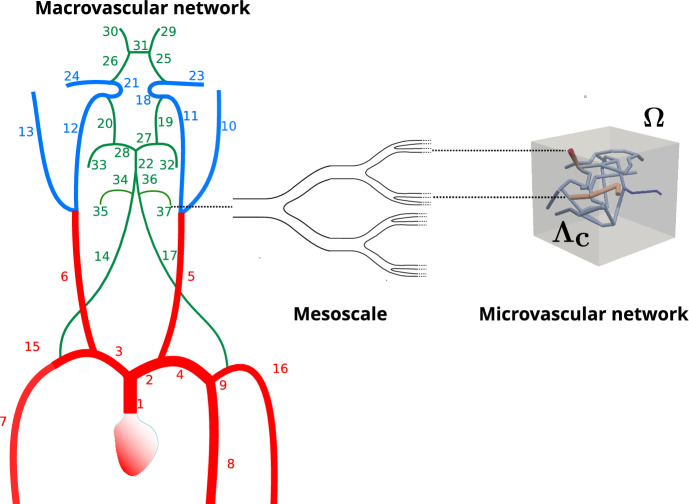
This figure gives an overview on the vascular systems that are considered for simulating blood flow from the heart to the neuro-glial-vascular unit. The macrovascular network and mesoscaled network are part of the arterial tree, while the microvascular network has to be assigned to the capillaries. By means of the macrovascular network, we determine the amount of blood volume transported towards the head in particular through the vertebral arteries. The mesoscaled network connects one of the vertebral arteries with the capillary network $\Lambda _c$ supplying our neuro-glial unit contained in $\Omega$

## Neuro-Glial-Vascular unit model

### Blood flow model

In order to determine the blood supply of a NGVU, we consider a capillary network contained in a block measuring $150 \times 160 \times 140~\mu \text {m}$. It is based on a three-dimensional vascular network reconstructed from scanning electron microscopy (SEM) data of the rat brain. The vascular structure, originally presented by (Motti et al. 1986), highlights the terminal vascular bed in the superficial cortex. The reconstruction and its application to oxygen transport simulations were detailed by (Secomb et al. 2000). This dataset, denoted by *brain99*, provides geometric and flow data for a vascular block $\Omega \subset {\mathbb {R}}^3$ measuring $150 \times 160 \times 140~\mu \text {m}$, including information on vessel radii, lengths, and Cartesian coordinates. The underlying microvascular casts originate from the superficial rat cortex characterized by (Motti et al. 1986).

The data set *brain99* is utilized as a representative microcirculation block for the DVC. Although *brain99* is derived from rat cortex, we use it as a morphological proxy for a small DVC microvascular unit to instantiate the NGVU; in this context it serves as a tractable approximation to human NGVU microarchitecture, with species differences noted. As a point of reference, *brain99* has also been used in particle-level transport studies on realistic vascular graphs to predict drug diffusion and delivery in rat brain microvasculature (Troendle et al. 2018). At the two largest capillary inlets, we couple the macro- and microcirculation to ensure a physiologically consistent transition of flow and pressure between arterial and capillary networks. For this purpose, we prescribe at the respective inlets flow rates derived from a model for blood flow in a network composed of larger arteries. The network, which is considered in this work can be seen in Fig. 3. It consists of the largest arteries attached to the heart, as well as the brachial and carotid arteries, and the circle of Willis. This network is enhanced by two arteries that branch out from the vertebral arteries (Vessel 35 and 37 in Figure 3). Moreover, the vertebral arteries are split into two parts, where the distal parts are considered as new vessels. The four additional vessels are labeled with the indices 34, 35, 36 and 37. In the remainder of this work, this network is referred to as a macrovascular network. We assume that $\Omega$ is supplied by Vessel 37. In order to connect $\Omega$, we use an averaged flow rate at the outlet of Vessel 37 as input for a simple model representing the vessel tree linking Vessel 37 and the capillary network $\Lambda _c$ contained in $\Omega$. This vessel tree comprises a part of the mesoscale within the vascular tree. An an output of the mesoscale model, we obtain two flow rates that are prescribed at the inlets of the vessels with the two largest radii (see Figure 3). All in all we obtain a multi-scale model for a flow path starting at the inlet of the aorta and ending in the NGVU contained in $\Omega$.

In the following, we describe the different components of our model: In a first subsection, a dimension-reduced model for flow in the macrovascular network is presented. Next, a flow model within the microvascluar network is described. Finally, a strategy for coupling both models via a model for the mesoscale is discussed.

#### Blood flow model for the macrovascular network

For the macrovascular network we use the one-dimensional model (1D) described in Čanić and Kim (2003); Hughes and Lubliner (1973) and (Carlo 2007, Chapter 2) on each vessel. Hence, we determine the vessel area $A\;\left[ \hbox {cm}^2 \right]$, and flow rate $Q\;\left[ \hbox {cm}^3\hbox {s}^{-1}\right]$ on the center line $\Lambda$ of a blood vessel with length $L\;\left[ \hbox {cm} \right]$. $\Lambda$ is parameterized by a variable $z\in \left[ 0,L \right]$. *A* and *Q* are solutions of a non-linear hyperbolic system of equations given by (Köppl and Helmig (2023), Section 3.2):

1 $$\begin{aligned}&\partial _t A + \partial _z Q = 0, \;z \in \left( 0,L \right) ,\;t>0, \end{aligned}$$

2 $$\begin{aligned}&\partial _t Q + \partial _z\left( \frac{Q^2}{A} \right) + \rho ^{-1}A \,\partial _z P = - 2 \cdot \left( {\bar{\gamma }}+2 \right) \cdot \frac{\mu \left( 2 \cdot R \right) }{\rho } \cdot \frac{Q}{A},\;z \in \left( 0,L \right) ,\;t>0, \end{aligned}$$

where $t\;\left[ \hbox {s}\right]$ is the time variable and $\rho = 1.028 \cdot 10^{-3} \;\hbox {g}\,\hbox {cm}^{-3}$ represents the fluid density. $\mu \;\left[ \hbox {Pa\;s}\right]$ is a function describing the viscosity depending on the vessel radius $R\;\left[ \hbox {cm}\right]$. The formula for this function can be found in Pries et al. (1996). ${\bar{\gamma }} = 9$ is a shape parameter for the flow profile (Carlo 2007, Cp. 2 & Sec. 6.1). The pressure is related to the vessels by a fluid-structure interaction model, which is known as the Young-Laplace equation Olufsen (1999); Toro (2016):

3 $$\begin{aligned} P = G_{0} \left( \sqrt{\frac{A}{A_{0}}} - 1 \right) . \end{aligned}$$

The parameter $G_0$ is given by:

4 $$\begin{aligned} G_{0} = \frac{\sqrt{\pi } \cdot h_{0} \cdot E}{\left( 1-\nu ^2\right) \cdot \sqrt{A_{0}}}. \end{aligned}$$

$h_0\,\left[ \hbox {cm}\right]$ is the wall thickness, $E\,\left[ \hbox {Pa}\right]$ the elastic modulus, $\nu = \tfrac{1}{2}$ is the Poisson ratio and the vessel area for $P=0$ is denoted by $A_0\,\left[ \hbox {cm}^2\right]$. The radius of $A_0$ is given by $R_0\,\left[ \hbox {cm}\right]$.

A table containing the values for *L*, $h_0$, *E* and $R_0$ can be found in (Alastruey et al. (2007), Table 1). In this table one can find the data for Vessel 1-33 apart from the lengths of Vessel 14 and 17. The missing data for Vessel 34 to 37 as well as Vessel 14 and 17 can be listed in Table 2.

**Table 2:** Parameters for Vessel 14 and 17 as well as Vessel 34 to 37

Vessel	$L\,\left[ \hbox {cm}\right]$	$R\,\left[ \hbox {cm}\right]$	$h_0\,\left[ \hbox {cm}\right]$	$E\,\left[ 10^6 \cdot \hbox {Pa}\right]$
14	9.866	0.136	0.034	0.8
17	9.866	0.136	0.034	0.8
34	4.930	0.136	0.034	0.8
35	6.000	0.080	0.034	0.8
36	4.930	0.136	0.034	0.8
37	6.000	0.080	0.034	0.8

At the bifurcations of the network, we enforce continuity of the total pressure and flow conservation. The heart beats are emulated by describing the flow rate at the inlet of the aorta (Vessel 1) by a half-sine wave. We denote this flow rate by $Q_{in}\;\left[ \hbox {cm}^3\hbox {s}^{-1}\right]$. It is given by the *T*-periodic extension of

5 $$\begin{aligned} Q_{in}\left( t\right) = {\left\{ \begin{array}{ll} Q_{\text {max}} \sin \left( \frac{\pi \cdot t}{0.3 \cdot T} \right) , & \text { for } 0 \le t \le 0.3 \cdot T \\ 0, & \text { for } 0.3 \cdot T < t \le T. \end{array}\right. } \end{aligned}$$

In case of our simulations, we set $T = 1.0\;\hbox {s}$ and $Q_{max }=485.0\;\hbox {cm}^3\hbox {s}^{-1}$. The influence of the omitted vasculature beyond the outlets is accounted for by a zero-dimensional (0D) Windkessel model Fritz et al. (2022). To calibrate the Windkessel parameters, a method described in (Köppl and Helmig (2023), Section 3.6.2) is used. All in all, this results in a 1D-0D coupled model.

#### Blood flow model for the microvascular network

Modeling blood flow in a capillary network, we use a stationary description relating the 1D pressures $p_1$ on the capillary centerlines $\Lambda _c$ with the 3D pressures $p_3$ in the surrounding space $\Omega$:

6 $$\begin{aligned} - R^2 \pi \;\partial _{s} ( K_{1} \; \partial _{s} p_1 ) + 2 \pi R L_{p}( p_1 - \Pi _{\Omega }{p}_3)&= 0&\text {on } \Lambda _c, \end{aligned}$$

7 $$\begin{aligned} -\nabla \cdot ( K_3 \nabla p_3 ) + L_p ( p_3 - \Pi _\Lambda p_1 ) \delta _\Gamma&= 0&\text {on } \Omega , \end{aligned}$$

where $$\Pi _\Lambda : \Lambda _c \rightarrow \Gamma $$ extends functions on the center line to functions on the cylinder boundary, $$\Pi _{\Omega }: \Omega \rightarrow \Lambda $$ averages functions on cylinder cross sections, and $$\delta _\Gamma $$ the Dirac distribution on the cylindrical vessel walls. By this, we obtain a 3D-1D coupled model. *R* describes the radius of the vessels and $$L_p$$ is the permeability of the vessel walls. Moreover  and $$K_3 $$ are the permeabilities of the vessel and the medium contained in $$\Omega $$ (see Köppl et al. (2020) for details). As in Section 3.1.1, the viscosity function $$\mu $$ is taken from Pries et al. (1996). This flow model yields flow velocities in the capillary network $$\Lambda _c$$ and $$\Omega $$, which are denoted by $$v_c$$ and $$v_\Omega $$. The velocities are computed as follows:

8 $$\begin{aligned} \begin{aligned} v_c&= -K_1 \partial _{s} p_1 \\ v_\Omega&= -K_3\nabla p_3. \end{aligned} \end{aligned}$$

Using these velocities, convection diffusion equations are formulate to model the transport of oxygen, nutrients and other substances within $\Lambda _c$ and $\Omega$. As in case of the flow model there is a 1D PDE for the transport in the capillary network and a 3D PDE for the corresponding processes in the surrounding medium. For further information, we refer to Köppl et al. (2020). At the boundary of $\Omega$ we set homogeneous Neumann boundary conditions. The boundary conditions for $\Lambda _c$ are discussed in the next subsection.

#### Coupling of macro- and microcirculation

To couple the macrocirculation with the microcirculation, we assume that the inlets in our capillary bed are connected to the outlet of Vessel 37 by a surrogate model for the arterial tree between the capillary network and the outlet of Vessel 37 (see Figure 3). As in Fritz et al. (2022), it is assumed that these arterial trees are symmetric. This means that every vessel bifurcates into two child vessels following Murray’s law Murray (1926a, 1926b), and additionally, both child vessels have the same radii. Vessel radii between two arbitrary bifurcation levels *l* and $l+1$ are thus related by

9 $$\begin{aligned} r_l = 2^{1/\gamma } r_{l+1}, \end{aligned}$$

where $\gamma \in \left[ 2,3.5\right]$. If *n* bifurcations are given, we $r_0$ and the final radius $r_n$ are related to each other as follows:

10 $$\begin{aligned} r_0 = 2^{n/\gamma } r_{n}. \end{aligned}$$

The number of levels in our arterial tree is thus given by

11 $$\begin{aligned} n = \log _2( r_0/r_n)^{\gamma }. \end{aligned}$$

Assuming that the flow $Q_l$ at level *l* is evenly divided at each bifurcation yields the relationship $Q_l = 2 Q_{l+1}$. This implies:

12 $$\begin{aligned} Q_0 = 2^n Q_n = \left( \frac{r_0}{r_n} \right) ^\gamma Q_n. \end{aligned}$$

Coupling the macro- and microcirculation, we replace $r_0$ by the initial radius of Vessel 37: $r_0 = R_{0,37}$, while $r_n$ is the radius of a capillary containing an inlet. In case of our capillary network $\Lambda _c$, we choose the vessels at the boundary with the two largest radii. The corresponding radii are denoted by $r_{c,i},\;i\in \left\{ 1,2\right\}$. To compute $Q_0$, we simulate blood flow within the macrovascular network starting with $A=A_0$ and $Q=0$ for each vessel. The simulation is stopped after the pressure and flow rate curves become periodic for each heart beat. If this is the case, we report the flow rate curve $Q_{37}$ at the outlet of Vessel 37 for one heart beat i.e. for a time interval $\left[ t,t+T\right]$ and compute the average of $Q_{37}$. Finally, we equate $Q_0$ and this average:

13 $$\begin{aligned} Q_0 = \frac{1}{T} \int _{t}^{t+T}Q\left( t\right) \;dt. \end{aligned}$$

By this, the flow rates $Q_{r_{c,i}}$ are given by:

14 $$\begin{aligned} Q_{r_{c,i}} = Q_0 \cdot \left( \frac{r_{c,i}}{R_{0,37}} \right) ^\gamma \end{aligned}$$

To ensure mass conservation, we distribute at the outlets of $\Lambda _c$ the flow rate

15 $$\begin{aligned} Q_{in,c} = Q_{r_{c,1}} + Q_{r_{c,2}} \end{aligned}$$

entering the network according to their section areas. Since we have on both inlets and outlets Neumann boundary conditions, we have to fix the pressure at some place of the network such that an average pressure of about $30\;\hbox {mmHg}$ in $\Lambda _c$ results. Otherwise the flow problem for $\Lambda _c$ would be ill-posed.

### Quadripartite synapse model

The quadripartite synapse model of the DVC incorporates the Glu-, GABA-, neurons and astrocytes. The neurotransmission involves Glu, GABA, AMPA, NMDA, GABAa and GABAb type receptors, as described in (Brazhe et al. 2023; Tewari and Majumdar 2012). The model encompasses a pre-synaptic bouton, a post-synaptic dendritic spine-head, SMCs, and a perisynaptic astrocytes that governs $\hbox {Ca}^{2+}$ dynamics within the synaptic bouton. The calcium dynamics subsequently influence Glu release in the SMCs. The model further accounts for Glu concentration within the SMCs, incorporating the process of Glu reuptake by astrocytes. We further incorporated GABA neurons which have similar dynamics to the Glu-neurons. In addition, NMDA, GABAa and GABAb receptors with similar dynamics as AMPA receptor are considered.

Neuronal activities have the ability to induce elevations in $\hbox {Ca}^{2+}$ within astrocytes (Fellin 2009; Porter and McCarthy 1996), which in turn increases the concentration of $\hbox {Ca}^{2+}$ in neighboring glial cells, including astrocytes, that express a wide range of receptors (Newman 2003). The activation of these receptors leads to an increase in astrocytic [$\hbox {Ca}^{2+}$] and the subsequent release of various transmitters such as Glu, GABA, d-serine, and ATP. The depletion of the releasable vesicle pool due to recent neurotransmitter release in response to the pre-synaptic AP results in short-term synaptic depression (Ling-Gang et al. 1999; Brazhe et al. 2023). The proposed model is inspired by the experimental work conducted in (Perea and Araque 2007), who utilized hippocampal slice preparations from immature Wistar rats. The computational framework presented here integrates various detailed biophysical models to emphasize distinct facets of neurons-astrocytes signaling.

#### Pre-synaptic neuronal action potential and bouton calcium dynamics

The generation of pre-synaptic AP trains is achieved using the Hodgkin-Huxley formalism (Hodgkin and Huxley 1952). Elevation of $\hbox {Ca}^{2+}$ concentration within the pre-synaptic bouton is modeled by incorporating both rapid influx, utilizing single protein properties (Erler et al. 2004), and slower dynamics through a modified Li-Rinzel model (Li and Rinzel 1994). The initiation of the AP occurs at the axon hillock of the pre-synaptic neurons. The generation of the pre-synaptic AP in computational models has traditionally been accomplished using the Hodgkin-Huxley framework (Nadkarni and Jung 2003; Volman et al. 2007). Given that the details of pre-synaptic AP generation are not the primary focus of the present study, we adopt the Hodgkin-Huxley model to simulate regular spiking and burst activity for simplicity, given by

16 $$\begin{aligned} \begin{aligned} C \frac{\textrm{d}V_{pre}}{\textrm{d}t} =&I_{app} - g_K n^4 (V_{pre}-V_K) - g_{Na}m^3 h (V_{pre}-V_{Na})-g_L (V_{pre}-V_L), \\ \frac{\textrm{d}x}{\textrm{d}t} =&\,\alpha _x (1-x)-\beta _x x, \qquad x\in \{n,m,h\}, \end{aligned} \end{aligned}$$

where $V_{pre}$ denotes the pre-synaptic membrane potential, and $I_{app}$ represents the applied current density. The conductances for potassium, sodium, and leak channels are given by $g_K$, $g_{Na}$, and $g_L$ respectively. Correspondingly, $V_K$, $V_{Na}$, and $V_L$ are the reversal potentials for potassium, sodium, and leak channels. The variables *m* denotes the sodium activation, *h* is the sodium inactivation, and *n* is defined as the potassium activation. The expressions for $\alpha _x$ and $\beta _x$ for $x=(m,h,n)$ are given by

17 $$\begin{aligned}&\alpha _n = \frac{0.01 (-V_{pre}-60)}{\exp \left( \frac{-V_{pre}-60}{10} \right) -1}, \quad &  \beta _n = 0.125 \exp \left( \frac{-V_{pre}-70}{80}\right) , \end{aligned}$$

18 $$\begin{aligned}&\alpha _m = \frac{0.1 (-V_{pre}-45)}{\exp \left( \frac{-V_{pre}-45}{10}\right) -1}, \quad &  \beta _m = 4\exp \left( \frac{-V_{pre}-70}{18} \right) , \end{aligned}$$

19 $$\begin{aligned}&\alpha _h = 0.07 \exp \left( \frac{-V_{pre}-70}{20}\right) , \quad &  \beta _h =\frac{1}{\exp \left( \frac{-V_{pre}-40}{10}\right) +1}. \end{aligned}$$

The AP initiated at the axon hillock of the pre-synaptic neurons propagates along the axon to the terminal without degradation, causing an elevation in cytosolic [$\hbox {Ca}^{2+}$]. This increase in intracellular [$\hbox {Ca}^{2+}$] is composed of two parts: [$\hbox {Ca}^{2+}$] resulting from the AP, denoted as $c_{fast}$, and [$\hbox {Ca}^{2+}$] originating from intracellular stores, denoted as $c_{slow}$, based on the kinetics speed. Thus, the total intracellular [$\hbox {Ca}^{2+}$], represented as $c_i$, is defined as

20 $$\begin{aligned} c_i = c_{fast}+c_{slow} \rightarrow \frac{\textrm{d}c_i}{\textrm{d}t} = \frac{\textrm{d}c_{fast}}{\textrm{d}t}+\frac{\textrm{d}c_{slow}}{\textrm{d}t}. \end{aligned}$$

The significant impact of rapid $\hbox {Ca}^{2+}$ kinetics on neurotransmitter release is well documented (Bollmann et al. 2000). For simplicity, the $\hbox {Ca}^{2+}$ influx through the plasma membrane is modeled via N-type channels (Weber et al. 2010). Immature cells, as described in (Perea and Araque 2007), are assumed in this study. The equation governing [$\hbox {Ca}^{2+}$] resulting from the AP follows a straightforward construction and degradation framework (Keener and Sneyd 2009), which can be expressed as

21 $$\begin{aligned} \frac{\textrm{d}c_{fast}}{\textrm{d}t} = \underbrace{-\frac{I_{Ca}A_{btn}}{z_{Ca}FV_{btn}}+J_{PM,leak}}_{construction} \underbrace{- \frac{I_{PM,Ca}A_{btn}}{z_{Ca}FV_{btn}}}_{destruction}, \end{aligned}$$

where $I_{Ca}$ represents the $\hbox {Ca}^{2+}$ current through an N-type channel, $A_{btn}$ is the bouton’s surface area, $z_{Ca}$ is the valence of the $\hbox {Ca}^{2+}$ ion, *F* is Faraday’s constant, and $V_{btn}$ is the bouton’s volume. Additionally, $I_{PM,Ca}$ denotes the current attributed to the electrogenic plasma membrane $\hbox {Ca}^{2+}$ ATPase, which expels excess $\hbox {Ca}^{2+}$ from the cell (Jensen et al. 2007). The function of this pump is described using a standard Michaelis-Menten type formalism (Erler et al. 2004). Furthermore, $J_{PM,leak}$ accounts for the positive leak of $\hbox {Ca}^{2+}$ from the extracellular space into the bouton, ensuring that the Michaelis-Menten pump does not reduce cytosolic $\hbox {Ca}^{2+}$ to zero (Blackwell 2005). The $\hbox {Ca}^{2+}$ current through the N-type channel is modeled at the single protein level (Erler et al. 2004) as

22 $$\begin{aligned} I_{Ca} = \rho _{Ca} m^2_{Ca} \underbrace{g_{Ca}(V_{pre}(t)-V_{Ca})}_{single~open~channel}, \end{aligned}$$

where $\rho _{Ca}$ represents the density of N-type channel proteins, determining the number of $\hbox {Ca}^{2+}$ channels present on the bouton membrane. The parameter $g_{Ca}$ denotes the conductance of a single N-type channel, while $V_{Ca}$ is the reversal potential for the $\hbox {Ca}^{2+}$ ion, given by the Nernst equation (Keener and Sneyd 2009) as

23 $$\begin{aligned} V_{Ca} = \frac{RT}{z_{Ca}F} \ln {\left( \frac{c_{ext}}{c_i^{rest}}\right) }, \end{aligned}$$

where *R* denotes the universal gas constant, *T* is the absolute temperature, $c_{ext}$ represents the extracellular $\hbox {Ca}^{2+}$ concentration, and $c_i^{rest}$ signifies the resting total intracellular [$\hbox {Ca}^{2+}$]. It is assumed that each N-type channel is composed of two gates, with $m_{Ca}$ indicating the probability of a single gate being open. Therefore, the probability of a single N-type channel being open, which requires both gates to be open, is given by $m^2_{Ca}$. The time evolution of this single-channel open probability follows a Hodgkin-Huxley type model. The steady-state value $m^\infty _{Ca}$, as determined according to (Ishikawa et al. 2005), fits the Boltzmann function to the whole-cell current of an N-type channel. The variable $m_{Ca}$ approaches its asymptotic value $m^\infty _{Ca}$ with a time constant $\tau _{m_{Ca}}$ expressed by means of an ordinary differential equation as

24 $$\begin{aligned} \frac{\textrm{d}m_{Ca}}{\textrm{d}t} = \frac{(m^\infty _{Ca}-m_{Ca})}{\tau _{m_{Ca}}}. \end{aligned}$$

Additional mathematical expressions for the parameters utilized in (21) are given by

25 $$\begin{aligned} I_{PM,Ca}&= v_{PM,Ca} \frac{c_i^2}{c_i^2 + K^2_{PM,Ca}}, \end{aligned}$$

26 $$\begin{aligned} J_{PM,leak}&= v_{leak} (c_{ext}-c_i), \end{aligned}$$

27 $$\begin{aligned} m^{\infty }_{Ca}&= \frac{1}{1+\exp {((V_{m_{Ca}}-V_m)/k_{m_{Ca}})}}, \end{aligned}$$

where $v_{PMCA}$ denotes the maximum plasma membrane $\hbox {Ca}^{2+}$ ATPase (PMCA) current density, determined through computer simulation, so that $c_i$ is maintained at its resting concentration. The second component of bouton $\hbox {Ca}^{2+}$, denoted as $c_{slow}$, is recognized for its essential role in short-term plasticity (Emptage et al. 2001). The release of $\hbox {Ca}^{2+}$ from the ER is primarily regulated by two types of receptors or $\hbox {Ca}^{2+}$ channels: the IP$_3$ receptor and the ryanodine receptor (Sneyd and Falcke 2005). In this context, the flow is considered to occur solely through the IP$_3$ receptor. The IP$_3$ required for the release of $\hbox {Ca}^{2+}$ from the ER is generated when Glu binds to metabotropic Glu-receptors, activating a G-protein-linked pathway to phospholipase C, which then cleaves phosphatidylinositol (4,5)-bisphosphate (PIP$_2$) to produce IP$_3$ and diacylglycerol. For modeling this slower $\hbox {Ca}^{2+}$ signaling process, an adapted Li-Rinzel model is employed (*cf*. (Tsodyks and Markram 1997)). Originally, the model assumes the total intracellular concentration $c_0$, is conserved and determines the ER $\hbox {Ca}^{2+}$ concentration, $c_{ER}$, following the relation

28 $$\begin{aligned} c_{ER} = \frac{c_0-c_i}{c_1}. \end{aligned}$$

Due to the presence of membrane fluxes, specifically $I_{Ca}$ and $I_{PMCA}$, the assumptions from the original Li-Rinzel model are not applicable in the current model. Moreover, the Li-Rinzel model also incorporates intracellular IP$_3$ concentration as a control parameter. To address these discrepancies, two additional equations governing the ER concentrations [$\hbox {Ca}^{2+}$] and [$\hbox {IP}_3$] have been incorporated into the Li-Rinzel model. The IP$_3$ production term has been made dependent on Glu to examine the effect of astrocytes $\hbox {Ca}^{2+}$ on $c_i$ (*cf*. (Nadkarni and Jung 2007)). The mathematical model governing the $c_{slow}$ dynamics is thus given by

29 $$\begin{aligned} \frac{\textrm{d}c_{slow}}{\textrm{d}t}&= -J_{chan}-J_{ER,pump}-J_{ER,leak}, \end{aligned}$$

30 $$\begin{aligned} \frac{\textrm{d}c_{ER}}{\textrm{d}t}&= - \frac{1}{c_1} \frac{\textrm{d}c_{slow}}{\textrm{d}t}, \end{aligned}$$

31 $$\begin{aligned} \frac{\textrm{d}p}{\textrm{d}t}&= v_g \frac{g_a^{0.3}}{k^{0.3}_g+g^{0.3}_a} - \tau _p (p-p_0), \end{aligned}$$

32 $$\begin{aligned} \frac{\textrm{d}q}{\textrm{d}t}&= \alpha _q (1-q) -\beta _q q. \end{aligned}$$

Here, $J_{chan}$ represents the $\hbox {Ca}^{2+}$ flux from the ER to the intracellular space through IP$_3$ receptors, while $J_{ER,pump}$ denotes the $\hbox {Ca}^{2+}$ flux being pumped from the intracellular space back into the ER. The variable $c_{ER}$ indicates the ER $\hbox {Ca}^{2+}$ concentration, $c_1$ is the ratio of ER volume to bouton volume, *p* represents the intracellular $\hbox {IP}_3$ concentration, $g_a$ signifies the Glu in the extra-SMCs, and *q* denotes the fraction of activated $\hbox {IP}_3$ receptors. These fluxes are described by the following expressions:

33 $$\begin{aligned} J_{chan}&= c_1 v_1 m^3_\infty n^3_\infty q^3 (c_i-c_{ER}), \end{aligned}$$

34 $$\begin{aligned} J_{ER,pump}&= \frac{v_3 c_i^2}{k^2_3 +c_i^2}, \end{aligned}$$

35 $$\begin{aligned} J_{ER,leak}&= c_1 v_2 (c_i -c_{ER}), \end{aligned}$$

with $m_\infty = \tfrac{p}{p+d_1}$, $n_\infty = \tfrac{c_i}{c_i + d_5}$, $\alpha _q = a_2 d_2 \tfrac{p+d_1}{p+d_3}$ and $\beta _q=a_2c_i$. The values of $v_1$, $v_2$, and $v_3$ are iteratively determined to ensure that $\hbox {Ca}^{2+}$ homeostasis is maintained within the cell and its organelles.

#### Glu- and GABA-Neuron dynamics in the bouton and smooth muscle cell

The dynamics of Glu-neuron interactions are analogous to those observed for GABA-neurons interactions, where Glu has an excitatory effect and GABA is an inhibitor (Petroff 2002). In the following sections, the mathematical model will be presented, focusing on the Glu-neurons system and its dynamics within the SMCs and the extra-SMCs. In the quadripartite synapse model, both Glu and GABA are incorporated, each with distinct kinetic constants. Glu release within the SMCs is represented as a two-step process: $\hbox {Ca}^{2+}$ binding to a synaptic vesicle sensor is described according to (Bollmann et al. 2000), while synaptic vesicle fusion and recycling follow the framework outlined in (Tsodyks and Markram 1997). The elevation of extra-synaptic Glu is also approached as a two-step process: the probability of synaptic-like micro-vesicle release is fitted using a modified model of (Bertram et al. 1996), and synaptic-like micro-vesicle fusion and recycling are again modeled following the Tsodyks-Markram model (Tsodyks and Markram 1997), incorporating recent empirical data from (Malarkey and Parpura 2011).

The AP waveforms result in a temporary rise in intracellular [$\hbox {Ca}^{2+}$], which subsequently triggers neurotransmitter release (Bollmann et al. 2000; Wang et al. 2009). Investigating the sensitivity of $\hbox {Ca}^{2+}$ sensors is challenging due to the small size of nerve terminals (Wang et al. 2009). It is generally assumed that a $\hbox {Ca}^{2+}$ concentration of at least 100 $\mu$M is required to activate a *low-affinity* $\hbox {Ca}^{2+}$ sensor (Nadkarni and Jung 2003). However, recent studies conducted in the giant calyx of Held terminal have demonstrated that an intracellular $\hbox {Ca}^{2+}$ concentration of approximately 10 $\mu$M is sufficient for Glu release (Bollmann et al. 2000). The kinetic model describing $\hbox {Ca}^{2+}$ binding to the $\hbox {Ca}^{2+}$ sensor is expressed by

36 $$\begin{aligned} X \overset{5 \alpha c_i}{\underset{\beta }{\rightleftharpoons }} X(c_i)_1 \overset{4 \alpha c_i}{\underset{2 \beta }{\rightleftharpoons }} X(c_i)_2 \overset{3\alpha c_i}{\underset{3 \beta }{\rightleftharpoons }} X (c_i)_3 \overset{2 \alpha c_i}{\underset{4 \beta }{\rightleftharpoons }} X(c_i)_4 \overset{\alpha c_i}{\underset{5 \beta }{\rightleftharpoons }} X(c_i)_5 \overset{\gamma }{\underset{\delta }{\rightleftharpoons }}X(c_i)^*_5, \end{aligned}$$

where the constants $\alpha$ and $\beta$ represent the $\hbox {Ca}^{2+}$ association and dissociation rates, respectively, while $\gamma$ and $\delta$ are $\hbox {Ca}^{2+}$-independent isomerization constants. The variable *X* denotes the $\hbox {Ca}^{2+}$ sensor of a synaptic vesicle without any bound $\hbox {Ca}^{2+}$ ions, with the index number indicating the number of bound $\hbox {Ca}^{2+}$ ions. The isomer $X(c_i)_5^*$ is the form of $X(c_i)_5$ that is prepared for Glu release. The fraction of docked vesicles ready for release, $f_r$, has been determined using dynamic Monte Carlo simulations (Fall et al. 2002) based on the kinetics described in (36), which depends on the state $X(c_i)_5^*$. This approach is necessary because the number of synaptic vesicles with five $\hbox {Ca}^{2+}$ ions bound cannot be estimated by the average vesicle pool due to the limited number of vesicles. The release of neurotransmitters spontaneously depends on the pre-synaptic $\hbox {Ca}^{2+}$ concentration (Bollmann et al. 2000; Emptage et al. 2001). The number of vesicles ready for spontaneous release, $p_r$, is assumed to follow a Poisson process (Nadkarni et al. 2008), which is given by the rate

37 $$\begin{aligned} \lambda (c_i) = a_3 \frac{1}{\left( 1+\exp {\left( \frac{a_1-c_i}{a_2}\right) }\right) }, \end{aligned}$$

that is iteratively adjusted to align with the frequency of spontaneous vesicle release in the presence of an astrocytes, typically ranging from 1 to 3 events per second (Kang et al. 1998). The vesicle fusion and recycling process is described by the Tsodyks-Markram model (Tsodyks and Markram 1997). To incorporate the dependency on $p_r$, a slight modification has been made to the Tsodyks-Markram model, yielding

38 $$\begin{aligned} \frac{\textrm{d}R}{\textrm{d}t}&= \frac{I}{\tau _{rec}} - f_r R, \end{aligned}$$

39 $$\begin{aligned} \frac{\textrm{d}E}{\textrm{d}t}&= - \frac{E}{\tau _{intact}}+f_r R, \end{aligned}$$

40 $$\begin{aligned} I&= 1 -R -E. \end{aligned}$$

Here, *R* represents the fraction of releasable vesicles within the bouton, *E* denotes the fraction of effective vesicles in the SMCs, and *I* signifies the fraction of inactive vesicles undergoing recycling. The variable $f_r$ can take values of 0, 0.5, or 1, corresponding to the number of vesicles ready for release, which are according to 0, 1, or 2. These values are determined either by the stochastic simulation of the kinetic model in (36) or by generating a Poisson random variable with the rate given by (37). The time constants for vesicle inactivation and recovery are denoted by $\tau _{inact}$ and $\tau _{rec}$, respectively. Once a vesicle is released, the vesicle release process remains inactive for a duration of ${6.34}\,\hbox {ms}$ in case of Glu and in case of GABA ${7.2}\,\hbox {ms}$ (Dobrunz et al. 1997).

Using Monte Carlo simulations of a central glutamatergic synapse, Franks et al. (Franks et al. 2002) demonstrated that glutamatergic signaling is spatially independent at these synapses. The Glu concentration within a bouton vesicle has been estimated to be $60\,\hbox {mM}$ (Danbolt 2001). Given that *E* represents the effective fraction of vesicles in the SMCs, the Glu concentration in the SMCs can be mathematically expressed as

41 $$\begin{aligned} \frac{\textrm{d}g}{\textrm{d}t} = n_v g_v E - g_c g, \end{aligned}$$

where *g* represents the Glu concentration in the SMCs, $n_v$ is the number of docked vesicles, $g_v$ denotes the vesicular Glu concentration, and $g_c$ is the rate of Glu clearance (Destexhe et al. 1998). Using this straightforward dynamic model, it is possible to estimate a range for the Glu concentration in the SMCs from 0.24 to ${11}\,\hbox {nM}$ (Danbolt 2001; Franks et al. 2002), as well as to capture the time course of Glu in the SMCs over approximately ${2}\,\hbox {ms}$ (Franks et al. 2002; Clements 1996). The governing Glu dynamics in the extra-SMCs is described by

42 $$\begin{aligned} \frac{\textrm{d}g_a}{\textrm{d}t} = n^v_a g_a^v E_a - g^c_a g_a, \end{aligned}$$

where $g_a$ is the Glu concentration in the extra-SMCs, $n^v_a$ represents the readily releasable pool of synaptic-like micro-vesicles, $g^v_a$ is the Glu concentration within each synaptic-like micro-vesicle and $g^c_a$ is the clearance rate of Glu from the SMCs due to diffusion and/or re-uptake by astrocytes. Furthermore, it is evident that the formula is similar to Equation (41). In this case, Glu acts on extra-synaptic metabotropic Glu receptors located on the pre-synaptic bouton. This input is used for the IP$_3$ production term in Equation (29). Unlike the densely packed synaptic vesicles of neurons, the synaptic-like micro-vesicles of astrocytes are less densely packed (Bezzi et al. 2004). Therefore, it is assumed that each synaptic-like micro-vesicle contains 60 mM of Glu and 20 mM of GABA (Montana 2006).

#### Astrocyte calcium dynamics and gliotransmitter release

The modulation of astrocytic $\hbox {Ca}^{2+}$ levels by Glu is described using an astrocytes-specific G-ChI model (*cf*. (De Pittà et al. 2009)). This model is referred to as the G-ChI model, based on the dependent variables and the Glu concentration parameter utilized. Within this framework, *G* signifies the Glu concentration in the SMCs, *C* is the astrocytic [$\hbox {Ca}^{2+}$], *h* represents the gating variable of IP$_3$ receptors, and *I* indicates astrocytic IP$_3$ concentration. The variable *g* is treated as a dynamic variable as described in (41). Within this model, the astrocytic $\hbox {Ca}^{2+}$ concentration, $c_a$, primarily depends on two parameters: the flux from the ER into the cytosol and the maximum pumping capacity of the saroplasmatic reticulum ATPase pump. It is established that $\hbox {IP}_3$ receptors are organized in clusters within astrocytes (Holtzclaw et al. 2002). The cluster size, $N_{IP3}$, is not precisely known and is assumed to be 20 in this context (*cf.* (Shuai and Jung 2002)). The stochastic Li-Rinzel model is employed here (*cf*. (Shuai and Jung 2002)), which is expressed as

43 $$\begin{aligned}&\begin{aligned} \frac{\textrm{d}c_a}{\textrm{d}t}&= (r_{Ca} m^3_\infty n^3_\infty h^3_a) (c_0 -(1+c_{1,a})c_a) - v_{ER} \frac{c_a^2}{c_a^2 +K^2_{ER}} \\&\quad + r_L (c_0 - (1+c_{1,a})c_a), \end{aligned} \end{aligned}$$

44 $$\begin{aligned}&\begin{aligned} \frac{\textrm{d}p_a}{\textrm{d}t}&= v_\beta \text {Hill} \left( g^{0.9}, K_R \left( 1+\frac{K_p}{K_R} \text {Hill} (C,K_\pi ) \right) \right) + \frac{v_\delta }{1 + \frac{p_a}{k_\delta }} \text {Hill}(c_a^2, K_{PLC\delta }) \\&\quad - v_{3K} \text {Hill}(c_a^4,K_D) \text {Hill} (p_a,K_3) -r_{5_{Pa}}p_a, \end{aligned} \end{aligned}$$

45 $$\begin{aligned}&\begin{aligned} \frac{\textrm{d}h_a}{\textrm{d}t}&= \alpha _{h_a} (1-h_a) - \beta _{h_a}h_a+G_h (t). \end{aligned} \end{aligned}$$

Equation (43) consists of three terms on the right-hand side. The first one represents the $\hbox {Ca}^{2+}$ flux from the ER into the intracellular space with $r_{Ca}$ denoting the maximal rate of the $\hbox {Ca}^{2+}$ flux from the $\hbox {IP}_3$ receptor, and $m^3_\infty n^3_\infty h^3_a$ together represent the opening probability of the IP$_3$ receptor cluster. The second term describes the rate at which $\hbox {Ca}^{2+}$ is removed from the intracellular space by the saroplasmatic reticulum ATPase pump with $v_{ER}$ representing the maximal rate of $\hbox {Ca}^{2+}$. The third term accounts for the leakage of $\hbox {Ca}^{2+}$ from the ER into the intracellular space. Here, $r_L$ is the maximal rate of $\hbox {Ca}^{2+}$ leak from the ER. The terms involved depict similarity to the production terms of $c_{slow}$ in (29), with the main distinction being that this model operates under a closed-cell assumption. Given this, an expression like (28) holds and can be represented using the astrocytic parameters as

46 $$\begin{aligned} c_{ER,a} = \frac{(c_0 -c_a)}{c_{1,a}}, \end{aligned}$$

which has the advantage that the astrocytic $\hbox {Ca}^{2+}$ flux terms can be represented completely in terms of cell parameters. The second equation (44) consists of four terms on the right-hand side. The first two terms represent the agonist-dependent and agonist-independent production of $\hbox {IP}_3$ and the last two incorporate the $\hbox {IP}_3$ degradation by $\hbox {IP}_3$-3K and $\hbox {IP}_3$-5P, respectively. In the last equation (45), $\alpha _{h_a}$ and $\beta _{h_a}$ denote the rates at which $h_a$ opens and closes, respectively, and $G_h (t)$ is a zero mean, uncorrelated, Gaussian white-noise term with a covariance function (*cf*. (Shuai and Jung 2002)), given by

47 $$\begin{aligned} \langle G_h (t), G_h (t') \rangle = \frac{\alpha _{h_a} (1-h_a) + \beta _{h_a} h_a}{N_{IP_3}} \delta (t-t'), \end{aligned}$$

where $\delta (t)$ is the Dirac-delta function, *t* and $t'$ are distinct times and the fraction denotes the spectral density (*cf*. (Coffey et al. 2004)). For astrocytic $\hbox {Ca}^{2+}$ oscillations, a model incorporating both amplitude and frequency modulation is used, as the coupling between IP$_3$ metabolism and CICR does not support amplitude-only modulation (De Pittà et al. 2009). The remaining expressions used in the equations (43) to (45) are

48 $$\begin{aligned} \begin{aligned} m_{\infty ,a}&= \text {Hill} (p_a, d_1), \\ n_{\infty ,a}&= \text {Hill}(c_a,d_5), \\ \text {Hill}(x^n,K)&= \frac{x^n}{x^n + K^n}, \\ \alpha _{h_a}&= a_2 d_2 \frac{p_a+d_1}{p_a + d_3}, \\ \beta _{h_a}&= a_2 c_a, \end{aligned} \end{aligned}$$

where the generic Hill function $\text {Hill}(x^n, K)$ is used for reactions, whose reaction velocity curve is not hyperbolic (Keener and Sneyd 2009). Various studies have shown that astrocytes release gliotransmitters in a $\hbox {Ca}^{2+}$-dependent manner (Fellin 2009; Montana 2006). The released gliotransmitters modulate synaptic plasticity through extra-synaptic NMDA receptors (Parpura et al. 1994; Parpura and Haydon 2000) and extra-synaptic metabotropic Glu-receptors (Perea and Araque 2007). The release mechanism of gliotransmitters from astrocytes, similar to that of neurons, occurs in a vesicular manner (Montana 2006; Bezzi et al. 2004). Studies indicate that the $\hbox {Ca}^{2+}$ dependence of Glu release from hippocampal astrocytes and found that the Hill coefficient for this process ranged between 2.1 and 2.7, indicating that at least two $\hbox {Ca}^{2+}$ ions are essential for gliotransmitter release (*cf*. (Parpura and Haydon 2000)). Consequently, the probability of vesicular fusion in response to mechanical stimulation and the size of the readily releasable pool of synaptic-like micro-vesicles in astrocytes was determined in (Malarkey and Parpura 2011). Based on the work of Bertram et al. (*cf*. (Bertram et al. 1996)), the model describing gliotransmitter release site activation requires that three $\hbox {Ca}^{2+}$ ions must bind to three independent gates or sites ($S_1-S_3$) to enable gliotransmitter release, which is expressed as

49 $$\begin{aligned} C_a + C_j \overset{k^+_j}{\underset{k^-_j}{\rightleftharpoons }} O_j, \qquad j\in (1,2,3), \end{aligned}$$

with $C_j, O_j$ as the closing and equal opening probabilities of gate $S_j$, and, $k_j^+, k_j^-$, as the opening and closing rates of the gate $S_j$, respectively. The temporal evolution of the open gate $O_j$ can be expressed as

50 $$\begin{aligned} \frac{\textrm{d}O_j}{\textrm{d}t} = k^+_j c_a - (k^+_j c_a + k^-_j ) O_j. \end{aligned}$$

The fraction of synaptic-like micro-vesicles, $f_r^a$, ready to be released is described by the product of the opening probabilities, $O_j$ with $j\in (1,2,3)$, of the three sites due to their physically independency. The dissociation constants of gates $S_1, S_2, S_3$ are ${108}\,\hbox {nM}$, ${400}\,\hbox {nM}$ and $800\,\hbox {nM}$. The time constants for gate closure, $1/k_j^-$, are $2.5\,\hbox {s}$, $1\,\hbox {s}$ and $100\,\hbox {ms}$. $S_1$ and $S_2$, as well as the time constants, are adapted from (Bertram et al. 1996), while the dissociation and time constant for gate $S_3$ is determined iteratively to match the experimental data from (Malarkey and Parpura 2011). When the synaptic-like micro-vesicle is ready to be released, the fusion and recycling process is modeled by the Tsodyks-Markram model (Tsodyks and Markram 1997), given by

51 $$\begin{aligned} \begin{aligned} \frac{\textrm{d}R_a}{\textrm{d}t}&= \frac{I_a}{\tau ^a_{rec}}- \Theta (c_a - c_a^{thresh})f_r^a R_a, \\ \frac{\textrm{d}E_a}{\textrm{d}t}&= - \frac{E_a}{\tau _{inact}^a} + \Theta (c_a - c_a^{thresh})f_r^a R_a, \\ I_a&= 1 - R_a - E_a. \end{aligned} \end{aligned}$$

In this context, $R_a$ denotes the fraction of readily releasable synaptic-like micro-vesicles within the astrocytes, $E_a$ represents the fraction of effective synaptic-like micro-vesicles in the extra-SMCs, and $I_a$ signifies the fraction of inactive synaptic-like micro-vesicles undergoing endocytosis or re-acidification. The term $\Theta$ is the Heaviside function, and $c_a^{thresh}$ represents the threshold of astrocytes [$\hbox {Ca}^{2+}$] required for activation of the release site (Parpura and Haydon 2000). The time constants for the inactivation and recovery of synaptic-like micro-vesicles are denoted by $\tau ^a_{inact}$ and $\tau ^a_{rec}$, respectively.

### Components of the neurovascular coupling model

To extend the quadripartite synapse model into a NGVU model, it is essential to incorporate smooth muscle and endothelial cell contraction, as well as vascular mechanisms, to achieve completeness. For this purpose, we employ the neurovascular coupling model developed in (Dormanns et al. 2015), with necessary modifications to address certain limitations in the original. For instance, the original model incorrectly assumes that astrocytes exhibit an AP, which is not supported by current understanding. Additionally, it considers potassium as the primary driving component in neurons and astrocytes, an assumption that has been updated in light of recent findings (Brazhe et al. 2023). The neurovascular unit model described in (Dormanns et al. 2015) comprises seven fundamental compartments: neurons, the synaptic cleft, astrocytes, the perivascular space, SMCs, ECs, and the arteriolar lumen. These compartments are interconnected through four subsystems: (1) the neuron/astrocyte subsystem, which includes the perivascular space and the synaptic cleft, (2) the smooth muscle cell/endothelial cell subsystem that couples these two cell types, (3) the arteriolar contraction subsystem, and (4) the arteriolar wall mechanical subsystem. The subsystems are modeled as having a triggering input and a subsequent output, which establishes connectivity and serves as a triggering input for other interconnected subsystems (Dormanns et al. 2015). Given the incorrect assumptions about the neuron/astrocyte subsystem in the original model, we include only the latter three subsystems in the NGVU model.

#### Smooth muscle and endothelial cell dynamics

The subsystem combining the smooth muscle cell and endothelial cell is based on the work in (Koenigsberger et al. 2006). In the original model of (Dormanns et al. 2015), an KIR channel at the interface between the smooth muscle cell and the perivascular space is included. The inputs for this subsystem are the KIR channel on the smooth muscle cell facing the perivascular space, which allows a flux of $\hbox {K}^+$ into the cytosol, and the influx of $\hbox {IP}_3$ into the endothelial cell mediated by luminal agonist P2Y receptors on the endothelial cell membrane (Dormanns et al. 2015). The biochemical behavior of the KIR channel is modeled and implemented based on experimental data from (Filosa et al. 2006) as

52 $$\begin{aligned} J_{KIR_i} = \frac{F_{KIR_i} g_{KIR_i}}{\gamma _i} (v_i - v_{KIR_i}), \end{aligned}$$

where the conversion parameter $\gamma _i$ is used to provide $J_{KIR_i}$ in the correct units of $\hbox {mV}\,\hbox {s}^{-1}$. The Nernst potential (in $\hbox {mV}$) is a function of $K_p$ and is expressed as

53 $$\begin{aligned} v_{KIR_i} = z_1 K_p - z_2, \end{aligned}$$

where $z_1$ and $z_2$ are derived by fitting a linear function according to (Filosa et al. 2006). The conductance of the KIR channel depends on both the membrane potential $v_i$ and $K_p$. The values of the constants are likewise based on the experimental data from (Filosa et al. 2006). The second input to the smooth muscle cell/endothelial cell subsystem is the generation of IP$_3$ in the endothelial cell due to the activation of membrane receptors by agonists in the arteriolar lumen. IP$_3$ mediates the $J_{IP_3}$ channel in both the endothelial cell and smooth muscle cell, located on the surface of the endothelial cell and saroplasmatic reticulum, allowing $\hbox {Ca}^{2+}$ to be released from the reticulum. For a given $\hbox {IP}_3$ concentration in the smooth muscle cell/endothelial cell subsystem, $\hbox {Ca}^{2+}$ fluctuations can occur due to the CICR process (Goldbeter et al. 1990; Koenigsberger et al. 2005). The production of $\hbox {IP}_3$ in endothelial cell is represented by the flux $J_{EC,IP_3}$. In this study, $\hbox {IP}_3$ production is constant but can be extended to a time-dependent function.

Physiologically, endothelial and SMCs are connected by hetero- and homocellular gap junctions that allow intercellular exchange of molecules and voltage. In the neurovascular unit model in (Dormanns et al. 2015), the endothelial and SMCs are modeled as a single subsystem, thus the homocellular exchange between cells is neglected. The linearized coupling functions for the heterocellular exchange of calcium, voltage, and $\hbox {IP}_3$ are given by

54 $$\begin{aligned} J_{Ca^{2+}_{cpl}}&= -P_{Ca^{2+}} ([Ca^{2+}]_i - [Ca^{2+}]_j), \end{aligned}$$

55 $$\begin{aligned} J_{V_{cpl}}&= -G_v (V_i - V_j), \end{aligned}$$

56 $$\begin{aligned} J_{IP_{3,{cpl}}}&= -P_{IP_3} ([IP_3]_i-[IP_3]_j), \end{aligned}$$

where the subscripts *i* and *j* correspond to endothelial and SMCs, respectively. Additionally, calcium buffering is incorporated to account for the fact that approximately 1% of the intracellular $\hbox {Ca}^{2+}$ is free (Parthimos et al. 1999).

#### Arteriolar contraction model

The contraction force is generated through the formation of cross-bridges between actin and myosin filaments, a process regulated by cytosolic $\hbox {Ca}^{2+}$. The model for the arteriolar contraction subsystem builds upon the foundational work of Hai and Murphy (Hai and Murphy 1989). The input signal for this model is the compartmental cytosolic $\hbox {Ca}^{2+}$ concentration in SMCs. Myosin exists in four potential states during this process: free non-phosphorylated cross-bridges (M), free phosphorylated cross-bridges (Mp), attached phosphorylated cross-bridges (AMp), and attached dephosphorylated latch-bridges (AM). The dynamics of the fraction of myosin in each state are described by the following four equations as

57 $$\begin{aligned} \frac{\textrm{d}[Mp]}{\textrm{d}t}&= K_4 [AMp] + K_1 [M] - (K_2 + K_3) [Mp], \end{aligned}$$

58 $$\begin{aligned} \frac{\textrm{d}[AMp]}{\textrm{d}t}&= K_3 [Mp] + K_6 [AM] - (K_4 + K_5) [AMp], \end{aligned}$$

59 $$\begin{aligned} \frac{\textrm{d}[AM]}{\textrm{d}t}&= K_5 [AMp] - (K_7 + K_6) [AM], \end{aligned}$$

60 $$\begin{aligned} {[}M]&= 1 - [AM] - [AMp] - [Mp], \end{aligned}$$

where the rate constants $K_n$ with $n=(1,\ldots ,7)$ control the phosphorylation and bridge formation processes. The $\hbox {Ca}^{2+}$ dependence of the cross-bridge model is governed by the rate constants $K_1$ and $K_6$. According to (Koenigsberger et al. 2006), the total phosphorylation of myosin is a function of the smooth muscle cell compartmental $\hbox {Ca}^{2+}$. Thus, $K_1$ and $K_6$ are defined by the constants characterizing the $\hbox {Ca}^{2+}$ sensitivity of calcium-activated phosphorylation of myosin as

61 $$\begin{aligned} K_1 = K_6 = \gamma _{cross} [Ca^{2+}]^3_i. \end{aligned}$$

The active stress in a smooth muscle cell is directly proportional to the fraction of attached cross-bridges, $F_r$, which serves as an input parameter for the mechanical subsystem. The fraction of attached cross-bridges is mathematically described as follows

62 $$\begin{aligned} F_r = \frac{[AMp]+[AM]}{\left( [AMp]+[AM]\right) _{max}}. \end{aligned}$$

$\left( [AMp] + [AM]\right) _{max}$ represents the maximum possible value of the sum of the concentrations of attached phosphorylated ([*AMp*]) and dephosphorylated ([*AM*]) cross-bridges. It is used for normalization, ensuring that $F_r$ is dimensionless and scaled between 0 and 1, which facilitates its use as an input parameter for the mechanical subsystem.

#### Visco-Elastic model of the arteriolar wall

The mechanical properties of the arterial wall are assumed as visco-elastic and are modeled following the Kelvin-Voigt approach. This model comprises a Newtonian damper and a Hookean elastic spring connected in parallel. The input signal, which corresponds to the active stress state of the smooth muscle cell in the circumferential direction, is the fraction of attached myosin cross-bridges, $F_r$, from the mechanical subsystem. The circumferential stress in the arterial wall, $\sigma _{\theta \theta }$, is given by the linear elastic part with the Young’s modulus *E*, and the viscous part with the viscosity $\eta$, as

63 $$\begin{aligned} \sigma _{\theta \theta } = E \epsilon _{\theta \theta } + \eta \frac{\textrm{d}\epsilon _{\theta \theta }}{\textrm{d}t}=\frac{R\Delta p}{h}, \end{aligned}$$

where $\epsilon _{\theta \theta }$ is the strain in the arterial wall, $\Delta p$ represents the transmural pressure, *R* denotes the vessel radius, and *h* is the vessel thickness. For simplicity, the wall thickness in this simulation is considered a constant fraction of the radius, with $h=0.1R$. The parameters of this subsystem are adapted from experimental data available in (Davis and Gore 1985). A linear function is employed to map the transition from the fully activated state to the fully relaxed state, applicable to radii between $10\,\upmu \hbox {m}$ and $30\,\upmu \hbox {m}$. The Young’s modulus as well as the initial radius are assumed to be continuous functions of $F_r$, with a linear interpolation between the active ($E_{act}$) and passive ($E_{pas}$) experimental data points provided in (Davis and Gore 1985), expressed as

64 $$\begin{aligned} E(F_r) = E_{pas} + F_r (E_{act}-E_{pas}). \end{aligned}$$

This approach can similarly be applied to represent the initial radius, yielding

65 $$\begin{aligned} R_0 (F_r) = R_{0,pas}+F_r(R_{0,act}-R_{0,pas}). \end{aligned}$$

Transforming the latter equations into a time-dependent expression for the vessel radius results in

66 $$\begin{aligned} \frac{\textrm{d}R}{\textrm{d}t} = \frac{R_{0,pas}}{\eta } \left( \frac{R\Delta p}{h}-E(F_r) \frac{R-R_0(F_r)}{R_0(F_r)}\right) . \end{aligned}$$

The final equation (66) describes the dynamic behavior of the vessel radius as a function of the transmural pressure, visco-elastic properties of the wall, and the activation state of SMCs via $F_r$. This expression expressed the connection between mechanical forces and active stress, allowing for a time-dependent simulation of arteriolar behavior under varying physiological conditions, providing a model for the dynamic regulation of blood flow and vessel radii in response to changes in transmural pressure and smooth muscle activation.

#### Influence of the nitric oxide signaling pathway

NO plays a fundamental role in neurovascular coupling by mediating the dynamic regulation of vascular tone in response to neuronal activity. As a potent vasodilator, NO is critical for linking cellular signaling processes to changes in arteriolar diameter and cerebral blood flow. Its high diffusivity and involvement in complex biochemical pathways necessitate its inclusion in the neurovascular coupling model to achieve a mechanistic understanding of the connection between neuronal activation, vascular responses, and metabolic demands (Dormanns et al. 2016). The NO signaling pathway model is based on (Dormanns et al. 2016), which is an addition to their previously introduced neurovascular unit model (*cf*. (Dormanns et al. 2015)). The corresponding implementation is available in ModelDB (accession 232956) (Kenny and David 2016; McDougal et al. 2017). The NO signaling pathway is mathematically described by production, diffusion and consumption terms in different cells, as well as the interaction of NO with other biochemical species and ion channel open probabilities (Dormanns et al. 2016).

**Fig. 4:**
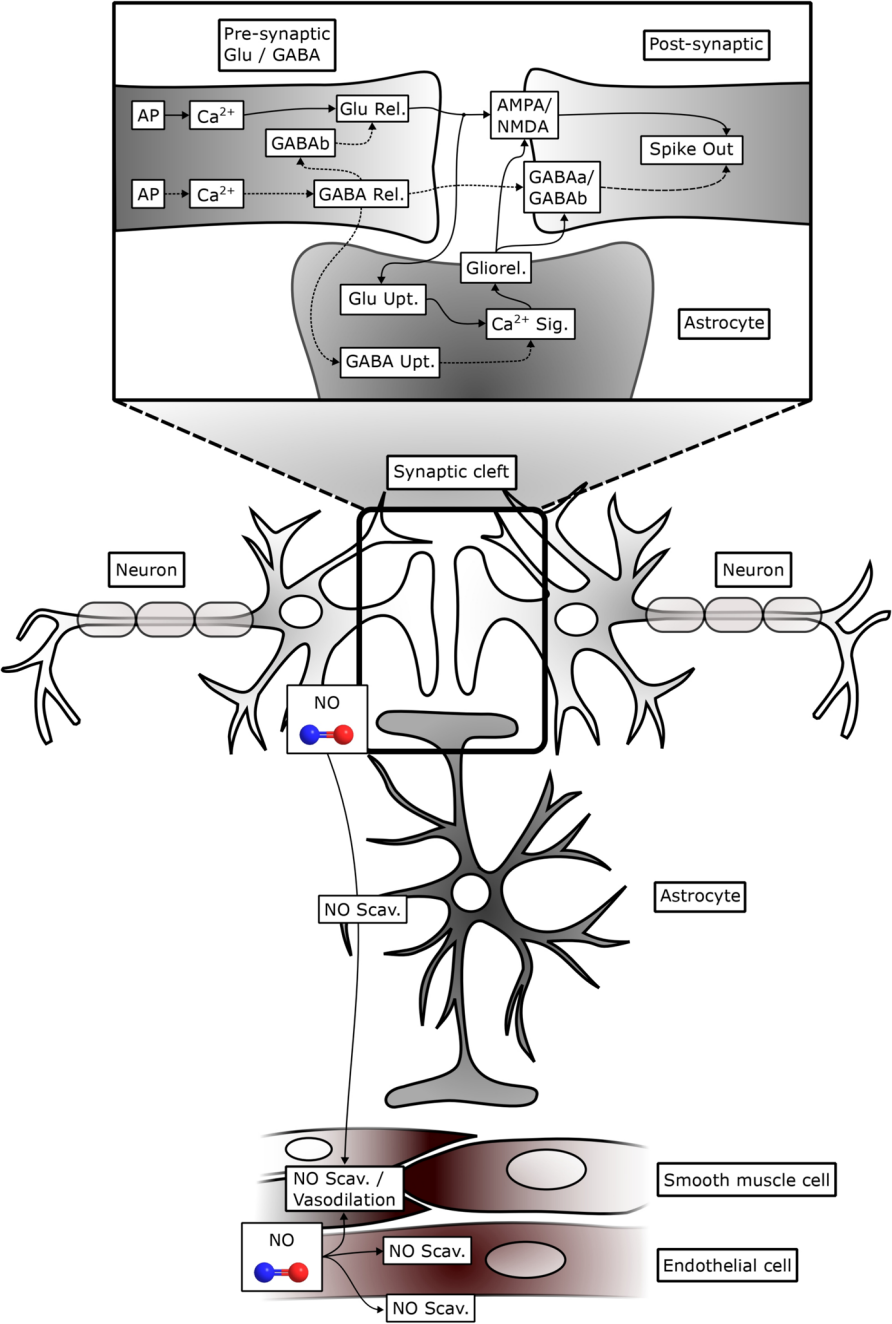
Components of the NGVU. Quadripartite synapse with pre-synaptic Glu/GABA neurons (top left), post-synaptic neuron (top right), astrocyte (center). Action potentials (AP) lead to Ca^2+^ influx, triggering Glu Rel/GABA Rel into the synaptic cleft. The astrocyte uptakes neurotransmitters (Glu Upt., GABA Upt.) and, via Ca^2+^ Sig., releases gliotransmitters (Gliorel). Endothelial and SMCs (bottom) regulate vasodilation through NO production and NO scavenging (Scav.). Abbreviations: AMPA/NMDA – excitatory receptors, GABA_a_/GABA_b_ – inhibitory receptors, Spike Out – post-synaptic spiking

The production of NO is driven by the two constitutive isoforms of nitric oxide synthases (NOS), neuronal nitric oxide synthase (nNOS) and endothelial nitric oxide synthase (eNOS)(Fleming and Busse 1999). The activation of both enzymes is mediated by intracellular $\hbox {Ca}^{2+}$ in the neurons and ECs, respectively. Additionally, eNOS is activated by blood flow-induced wall shear stress in cerebral arterioles (Joannides et al. 1995). Due to its high diffusion coefficient, NO rapidly diffuses into other compartments, as demonstrated by experimental data (Malinski et al. 1993) and kinetic simulations (Lancaster Jr 1994). When NO reaches the smooth muscle cell, it interacts with intracellular enzymes to regulate smooth muscle cell relaxation (Yang et al. 2005). The dynamics of NO in the various compartments are described using mass balance equations. The concentration of NO in each domain is determined by the balance between production, intracellular consumption, and diffusion to and from other compartments. Fig. 4 graphically illustrates the NO signaling pathway.

The rate of NO production is determined by the concentration of activated NO synthases. The essential biochemical substrates for this process include L-arginine (L-Arg), $\hbox {O}_2$, and nicotinamide adenine dinucleotide phosphate (hydrogen) (NADPH) (Chen and Popel 2006), with L-Arg serving as the crucial nitrogen donor (Forsythe et al. 2001). During the reaction, L-Arg is converted into L-citrulline, producing producing nicotinamide adenine dinucleotide phosphate $\hbox {(NADP)}^+$, water, and NO in the process. This reaction involves a five-electron oxidation that occurs in two distinct steps, and the overall stoichiometric equation is given by

67 $$\begin{aligned} \text {L-Arg} + 2\text {O}_2 + \frac{2}{3} \text {NADPH} \overset{NOS}{\longrightarrow }\ \text {L-Citrulline} + \frac{2}{3} \text {NADP}^+ + 2\text {H}_2\text {O} + \text {NO}. \end{aligned}$$

Several biomolecule cofactors, including flavin mononucleotide, flavin adenine dinucleotide, and tetrahydrobiopterin, are required for the reaction. The constitutive NOS isoforms, nNOS and eNOS, are considered the primary producers of NO and play a crucial role in maintaining homeostasis (Fleming and Busse 1999). Consequently, in this model, NO production is limited to neurons and ECs, with the production rates in astrocytes ($p_{NO,k}$) and SMCs ($p_{NO,i}$) set to zero. In neurons, the synthesis of NO is catalyzed by nNOS, which is activated in response to glutamate-induced calcium influx into the post-synaptic neurons. The production of NO in neurons depends on the concentration of activated nNOS and is further constrained by the availability of $\hbox {O}2$ and L-Arg (Chen and Popel 2006). This relationship is expressed mathematically through the maximum neuronal production rate, *p* *max*, as

68 $$\begin{aligned} \begin{aligned} p_{NO,n}&= p_{max} \frac{[O_2]_n}{K_{m,O2,n}+[O_2]_n} \frac{[L-Arg]_n}{K_{m,L-Arg,n}+[L-Arg]_n}, \\ p_{max}&= V_{max,NO,n} [nNOS_act]_n. \end{aligned} \end{aligned}$$

The maximum catalytic rate of nNOS, denoted as $V_{max,NO,n}$, along with the neuronal concentrations of O$_2$ and L-Arg, $[O_2]_n$ and $[L-Arg]_n$, respectively, and the Michaelis constants $K_{m,O2,n}$ and $K_{m,L-Arg,n}$, define the reaction kinetics (*cf*. (Chen and Popel 2006)). The activation of nNOS in the neurons is induced by the neurotransmitter Glu in response to neuronal activation. In a chemical synapse, when an AP arrives at the presynaptic neuronal axon terminal, Glu is released from vesicles into the synaptic cleft. This Glu then binds to receptors on the dendrite of the postsynaptic neurons and is subsequently cleared from the synaptic cleft through diffusion and hydrolysis (*cf*. (Dormanns et al. 2016; Brazhe et al. 2023; Tewari and Majumdar 2012)), as described in the quadripartite synapse model in Section 3.2.2.

Since the neuronal NOS is often co-localized with ionotropic NMDA-receptors (Mayer and Hemmens 1997; Förstermann et al. 1998), which are receptor complexes including transmembrane ion channels in the neurons that are opened or closed in response to the binding of Glu (Benarroch 2006). There are two different types of NMDA-receptors, NR2A and NR2B, which show different opening probabilities, *w*, which depend on the neuronal Glu concentration using Michaelis-Menten kinetics to meet the experimental data from (Santucci and Raghavachari 2008). Mathematically, the opening probability *w* is described by

69 $$\begin{aligned} w_{NR2,i} = \frac{[Glu]_{sc}}{K_{m,i}+[Glu]_{sc}}, \qquad i \in \{A,B\}, \end{aligned}$$

where $K_{m,A}$ and $K_{m,B}$ are the fitted Michaelis constants. Given that NMDA receptors are highly permeable to $\hbox {Ca}^{2+}$ ions (*cf*. (Jahr and Stevens 1993)), the glutamate-induced $\hbox {Ca}^{2+}$ flux is incorporated into the model. The total calcium current, $I_{Ca,tot}$, is expressed as

70 $$\begin{aligned} \begin{aligned} I_{Ca,tot}&= I_{Ca} (0.63 w_{NR2,A}+11 w_{NR2,B}) \\ I_{Ca}&= \frac{4v_nG_M(P_{Ca}/P_M) ([Ca^{2+}]_{ex}/[M])}{1+\exp (\alpha _v(v_n+\beta _n))} \frac{\exp (2v_n F/(R_{gas}T))}{1-\exp (2v_nF/(R_{gas}T))}, \end{aligned} \end{aligned}$$

where $I_{Ca}$ is the neuronal inward calcium current, *F* is Faraday’s constant, $v_n$ is the neuronal membrane potential, $G_M$ is the conductance, and $P_{Ca}/P_M$ is the ratio of NMDA receptor permeabilities to $\hbox {Ca}^{2+}$ and monovalent ions. Additionally, the model accounts for the external calcium concentration $[Ca^{2+}]_{ex}$, the concentration of monovalent ions [*M*], and the intra- and extracellular translation factors $\alpha _v$ and $\beta _v$. The temperature *T* and the universal gas constant $R_{gas}$ are also included. The estimated arrival of 0.63 NR2A- and 11 NR2B-NMDA-receptors, on average per synapse, is based on measurements from (Santucci and Raghavachari 2008). In the endothelial cell, NO production is facilitated by the constitutive enzyme isoform eNOS, whose catalytic activity depends on the availability of O$_2$ and L-Arg. The endothelial NO production can be described by

71 $$\begin{aligned} p_{NO,j} = V_{max,NO,j} [eNOS_{act}]_j \frac{[O_2]_j}{K_{m,O2,j} + [O_2]_j} \frac{[L-Arg]_j}{K_{m,L-Arg,j}+[L-Arg]_j}. \end{aligned}$$

The maximal activity of eNOS is regulated by the intracellular calcium concentration and is further influenced by wall shear stress, which arises from blood flow through the perfusing arteriole. Wall shear stress activates a signaling cascade involving phosphatidylinositol 3-kinase and the serine/threonine-specific protein kinase, which phosphorylates eNOS (Chen and Popel 2006; Comerford et al. 2008; Dormanns et al. 2016). The elastic strain energy stored within the vessel membrane, as well as the quantification of exogenous $\hbox {Ca}^{2+}$ entry via shear stress-gated ion channels, is detailed in (Comerford et al. 2008). The strain energy stored in the membrane, $F_{wss}$, and the strain energy density, $W_{wss}$, are described mathematically in (Wiesner et al. 1997). Wall shear stress, $\tau _{WSS}$, represents the frictional force per unit area and establishes a positive feedback mechanism: increased wall shear stress enhances NO production by ECs, inducing vasodilation and further modifying the shear stress. The wall shear stress $\tau _{WSS}$ in the arteriolar wall depends on the regional CBF, *Q*, which is modeled using the Hagen–Poiseuille flow assumption for cerebral arterioles.

The diffusion of NO, denoted as $d_{NO,m}$, can be derived from Fick’s second law of diffusion, which characterizes the diffusion of a substance over time and space. To simplify the diffusion formulation, the Einstein-Smoluchowski equation is employed, describing the characteristic time required for NO to diffuse a certain distance from the center of one cell to another. The compartment model outlines the diffusion between different domains but does not account for the amount of NO released into the lumen and subsequently scavenged by reactions with hemoglobin. The diffusion term of a linear diffusion is given by

72 $$\begin{aligned} d_{NO,m}&= \frac{[NO]_{out} - [NO]_{in}}{\tau _{\Delta x, m}} , \qquad m \in \{n,a,s,e\}, \end{aligned}$$

73 $$\begin{aligned} \tau _{\Delta x,m}&= \frac{(\Delta x_m)^2}{2D_{c,NO}}, \end{aligned}$$

where $m \in \{n,a,s,e\}$ denotes the cell indices for neurons, astrocytes, smooth muscle and ECs, respectively, $\tau _{\Delta _x}$ represents the characteristic time required for NO to diffuse over a specific distance $\Delta x_m$ from the center of one cell to another. For example, the distances between the centers of the neurons and astrocytes is roughly $25\,\upmu \hbox {m}$ (Dormanns et al. 2016; Kavdia et al. 2002).

The consumption of NO is denoted as $c_{NO,m}$. As a free radical, NO readily reacts with biochemical species containing unpaired electrons, such as molecular O$_2$, superoxide anions, and metals (Mayer and Hemmens 1997). NO is scavenged in the cytosol of all cell types through which it diffuses. The mathematical expression for the scavenging term across all model compartments is given by:

74 $$\begin{aligned} c_{NO,m} = k_m [NO]_m C_m , \qquad m \in \{n,a,e\}, \end{aligned}$$

where $C_m$ is the concentration of reactive species in the cell type, and $k_m$ is the reaction rate constant (Kavdia et al. 2002). In the smooth muscle cell, NO affects the contraction mechanism and the open probability of the large conductance $\hbox {Ca}^{2+}$-dependent $\hbox {K}^+$ channel (BK) channel via its second messenger, cGMP. Additionally, NO activates soluble guanylyl cyclase, an enzyme that catalyzes the formation of cGMP. The kinetics of these processes are described in (Yang et al. 2005).

### Integration of synapse and vascular components in NGVU

To integrate the quadripartite synapse and vascularization models into a coherent NGVU model, the interaction points between subsystems must be carefully addressed. First, the neurons/astrocytes subsystem from the original neurovascular unit model in (Dormanns et al. 2015) is extended with the quadripartite synapse model, while the vascularization components retain the smooth muscle cell/endothelial cell model, as well as the arteriolar contraction and mechanical wall models. Specifically, the astrocytic $\hbox {Ca}^{2+}$ released over time is incorporated into the sarcoplasmic reticulum as an uptake process. The $\hbox {Ca}^{2+}$ flux into the sarcoplasmic reticulum is modeled a

75 $$\begin{aligned} J_{SR_{uptake,i}}=B_i \frac{[Ca^{2+}]^2_i}{c^2_{bi}+[Ca^{2+}]^2_i}, \end{aligned}$$

where $[Ca^{2+}]_i$ represents the time-dependent $\hbox {Ca}^{2+}$ dynamics in astrocytes as defined by the quadripartite synapse model. The $\hbox {Ca}^{2+}$ release through the sarcoplasmic reticulum leak channel is driven by IP$_3$ generation. The initial input to the smooth muscle cell/endothelial cell subsystem through the KIR channel is retained in the NGVU model. Fig. 4 provides an overview of the complete NGVU model, integrating the quadripartite synapse and vascularization components, along with the relevant channels and fluxes.

To integrate the gasotransmitter NO into the NGVU model, Yang et al. (Yang et al. 2005) proposed two pathways through which NO can induce local vasodilation of arterioles in the brain. The first pathway involves an indirect influence on the smooth muscle cell contractile system, which is governed by the formation of cross-bridges between actin and myosin filaments, as initially described by Hai and Murphy (Hai and Murphy 1988). Furthermore, cGMP exerts a regulatory effect on the myosin dephosphorylation process, altering the rate constants for dephosphorylation as described by Yang et al. (Yang et al. 2005), represented by

76 $$\begin{aligned} R_{cGMP}&=\frac{[cGMP]_i^2}{K^2_{m,mlcp}+[cGMP]^2_i}, \end{aligned}$$

77 $$\begin{aligned} K_{2c}&= K_{5c} = \delta _i (k_{mlpc,b}+k_{mlpc,c}R_{cGMP}), \end{aligned}$$

where $K_{m,mlcp}$ denotes the Michaelis constant, $k_{mlpc,b}$ is the basal myosin light-chain dephosphorylation rate constant, $k_{mlpc,c}$ is the first-order rate constant for cGMP-regulated myosin light-chain dephosphorylation, and $\delta _i$ is a constant fitted to experimental data (*cf*. (Yang et al. 2005; Hai and Murphy 1988)). The second messenger of NO, cGMP, modifies the rate constants for the dephosphorylation of *Mp* to *M* and *AMp* to *AM* via myosin light-chain phosphatase (Yang et al. 2005). The second pathway concerns the open probability, $w_i$, of the BK channel in SMCs, which is a function of the membrane potential, $v_i$, and is shifted to the left in the membrane potential space by cGMP (*cf*. (Stockand and Sansom 1996)). Instead of using the open probability of the BK channel as described by Koenigsberger et al. (Koenigsberger et al. 2006), the influence of NO is incorporated as a cGMP-dependent modification of the open probability, $w_i$. Mathematically, this is expressed as

78 $$\begin{aligned} c_{w,i} = \frac{1}{\epsilon _i + \alpha _i \exp (\gamma _i[cGMP]_i)}, \end{aligned}$$

where $\epsilon _i$, $\alpha _i$, and $\gamma _i$ are translation factors calibrated to match the observed data from Stockand and Sansom (Stockand and Sansom 1996). Furthermore, to fully integrate the NO model into the NGVU model, we use the Glu in the synaptic cleft, provided by the quadripartite synapse model.

## Numerical simulation results

**Fig. 5:**
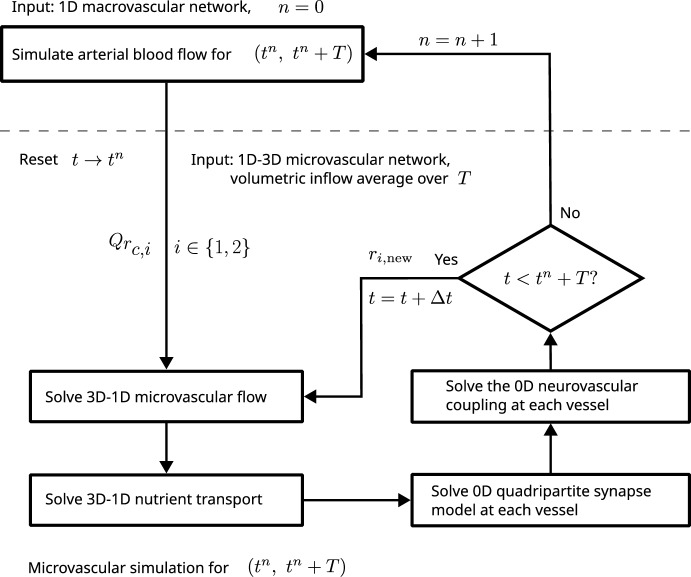
Schematic of the simulation workflow during one heartbeat period (*T*), illustrating the sequential coupling between the 1D macrovascular network and the neurovascular-coupled 3D-1D-0D microvascular network. For additional algorithmic details on coupling among 0D, 1D, and 3D components, see (Köppl et al. 2020; Fritz et al. 2022)

The following sections present the computational results of our integrated neuro-glial-vascular coupling model. The findings are organized to provide a detailed examination of the macrocirculation dynamics, neurotransmitter release, neuronal spiking activity, astrocytic signaling, neurovascular responses, and microcirculation within a representative unit of the DVC. Each subsection highlights specific aspects of the modeled system, emphasizing the interactions between neuronal, astrocytic, and vascular components and their implications for blood flow regulation and metabolic support in the DVC. These results validate the framework and provide mechanistic insights into the physiological processes underlying neurovascular coupling. In all subsequent cellular-scale (0D) analyses, the displayed results are exemplary for the capillary vessel that receives the prescribed arterial inflow. As shown schematically in Figure 5, the simulation workflow couples the macrocirculation and neurovascular microcirculation over a single heartbeat period (*T*). A brief description of the numerical solver for the 3D-1D coupled problems can be found in (Köppl et al. (2020), Section 2.6), while (Fritz et al. (2022), Section 6) contains information on the numerical methods that are used to simulate arterial blood flow. For the 0D cellular-scale components, we outline the model structure and numerical integration setup. The implementation and model parameters are available in our public GitHub repository linked in the Data Availability statement.

### Macrocirculation dynamics

**Fig. 6:**
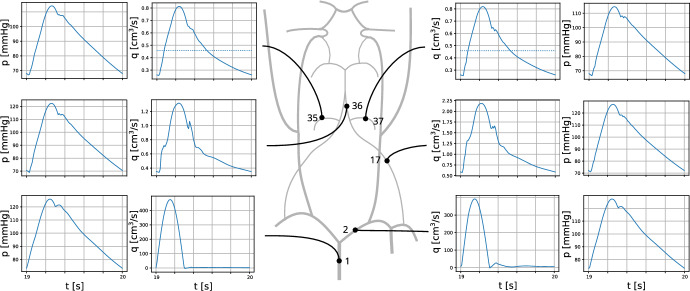
Flow and pressure in our Circle-of-Willis close to the coupling point with our microcirculation model

Figure 6 depicts the flow and pressure in our 1D-0D coupled model described in Section 3.1.1 at some sample points on the path from the aorta to the vessels that are coupled with the 3D-1D coupled microcirculation model (see Section 3.1.2). The average flows $Q_0$ used in the coupling are indicated by dotted lines. For the given setting assuming $\gamma = 3$ in (12) we obtain:

$$\begin{aligned} Q_0 \approx 0.46 \;\frac{\hbox {cm}^3}{\hbox {s}}. \end{aligned}$$

Based on this value we have for $\Lambda _c$ the following inflow rates:

$$\begin{aligned} Q_{r_{c,1}} \approx 8.19 \times 10^{-8} \frac{\hbox {cm}^{3}}{\hbox {s}} \text { and } Q_{r_{c,2}} \approx 5.75 \times 10^{-8} \frac{\hbox {cm}^{3}}{\hbox {s}}. \end{aligned}$$

Using the radii of the inlets ($r_{c,1} = 4.5 \cdot 10^{-4}\;\hbox {cm}$ and $r_{c,2} = 4.0 \cdot 10^{-4}\;\hbox {cm}$), we obtain the following flow velocities:

$$\begin{aligned} v_{r_{c,1}} \approx 0.13 \; \frac{\hbox {cm}}{\hbox {s}} \text { and } v_{r_{c,2}} \approx 0.11 \; \frac{\hbox {cm}}{\hbox {s}} . \end{aligned}$$

According to Ivanov et al. (1981) the flow velocity in brain capillaries varies between $0.05\;\frac{\hbox {cm}}{\hbox {s}}$ and about $0.15\;\frac{\hbox {cm}}{\hbox {s}}$. Thus, our simulation results are within a reasonable range.

The pressure curves in our macrovascular network exhibit the typical shape of pressure curves in larger arteries. It can be observed that the pressures are within a resonable range of $70\;\hbox {mmHg}$ (diastolic pressure) to about $125\;\hbox {mmHg}$ (systolic pressure) Heiss (1983). During the systole we have a strong increase and during the diastole a slow decay is seen. At the interface of both phases there is a characteristic kink caused by the closing of the aortic valve (Sidebotham (2007), Chapter 1, Figure 1.3). Due to the Windkessel effect caused by the models attached to the outlets of the macrovascular network the flow rates do not fall back to zero apart from the aorta (Vessel 1 and 2) Alastruey et al. (2007). This ensures a constant blood supply of the organs linked to the different outlets.

### Neurotransmitter release and synaptic activation

**Fig. 7:**
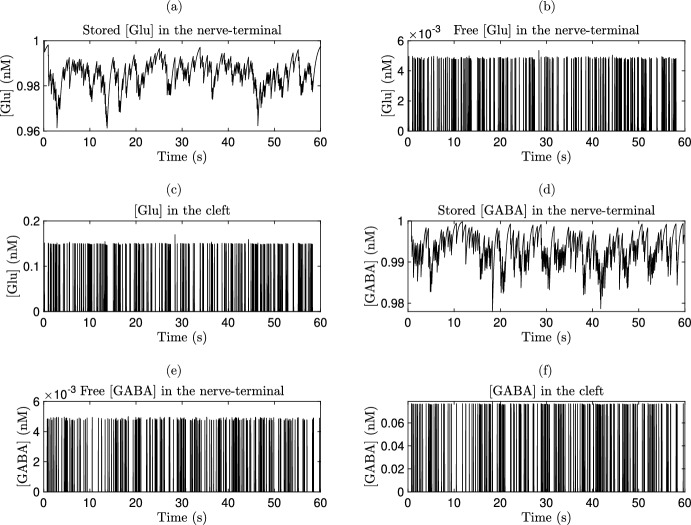
Dynamics of neurotransmitter release in a representative 0D quadripartite synapse system associated with one vessel. **(a)** Stored glutamate [Glu] in the nerve terminal, **(b)** Free glutamate [Glu] in the nerve terminal, **(c)** Glutamate [Glu] in the synaptic cleft, **(d)** Stored GABA in the nerve terminal, **(e)** Free GABA in the nerve terminal, and **(f)** GABA in the synaptic cleft

Figure 7 illustrates the dynamics of neurotransmitter release and synaptic activation in a representative 0D quadripartite synapse system associated with a single blood vessel. These results highlight the temporal variations in the concentrations of Glu and GABA within the nerve terminals and synaptic cleft, which serve as critical inputs to the neuro-glial-vascular coupling model.

In the case of glutamate, stored Glu in the nerve terminal (Figure 7a) gradually decreases due to synaptic activation, leading to the release of free Glu within the terminal (Figure 7b). A corresponding increase in glutamate concentration in the synaptic cleft is observed (Figure 7c), representing effective neurotransmitter signaling. Similarly, stored GABA (Figure 7d) transitions into free GABA (Figure 7e) and is subsequently released into the synaptic cleft (Figure 7f). These dynamics reflect the interplay of excitatory (Glu) and inhibitory (GABA) neurotransmitter systems, which are fundamental to maintaining synaptic balance and initiating downstream cellular responses.

**Fig. 8:**
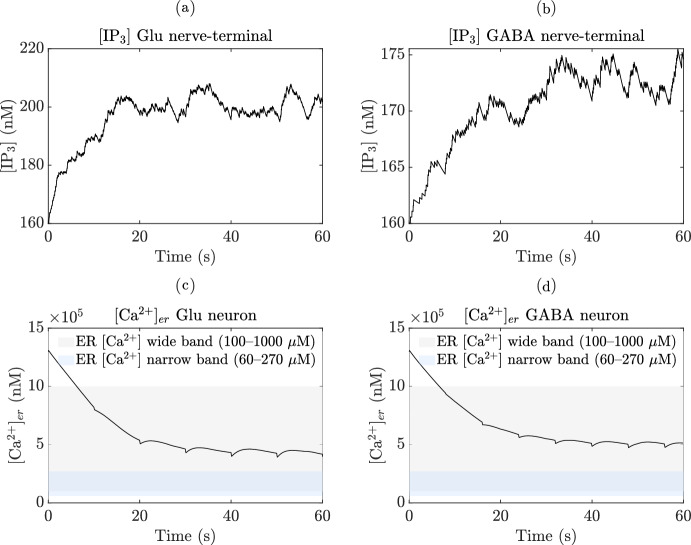
Dynamics of signaling molecules in the neuro–glial system for a representative quadripartite synapse system. **(a)** [$\hbox {IP}_3$] in the glutamate nerve terminal; **(b)** [$\hbox {IP}_3$] in the GABA nerve terminal; **(c)** [$\hbox {Ca}^{2+}$] concentration in the ER of the glutamatergic neuron; and **(d)** [$\hbox {Ca}^{2+}$] in the ER of the GABA neuron. The lower panels (c-d) include shaded ER [$\hbox {Ca}^{2+}$] reference bands: a broad range of 100–1000 $\mu \hbox {M}$ and a more constrained range of 60–270 $\mu \hbox {M}$, consistent with calibrated ER luminal measurements and contemporary reviews (Schulte and Blum 2022; Solovyova et al. 2002)

In addition to the neurotransmitter dynamics, Figure 8 illustrates the temporal evolution of key signaling molecules, including inositol trisphosphate ([$\hbox {IP}_3$]) and calcium ion ([$\hbox {Ca}^{2+}$]) concentrations. The [$\hbox {IP}_3$] dynamics in the glutamate nerve terminal (Figure 8a) and the GABA nerve terminal (Figure 8b) regulate transmitter release via downstream mobilization of intracellular $\hbox {Ca}^{2+}$. The endoplasmic-reticulum (ER) store dynamics in glutamatergic and GABAergic neurons (Figure 8c–d) are shown together with shaded reference bands that anchor the simulations to experimental measurements: a neuronal ER [$\hbox {Ca}^{2+}$] band of 60–270 $\mu$M based on calibrated recordings in sensory neurons (Solovyova et al. 2002), and a broader ER [$\hbox {Ca}^{2+}$] range of 100–1000 $\mu$M summarizing values reported across preparations and measurement methods (Schulte and Blum 2022). These experimentally grounded bands provide a concise visual check that the simulated trajectories fall within physiologically reported regimes.

### Neuronal spiking activity

**Fig. 9:**
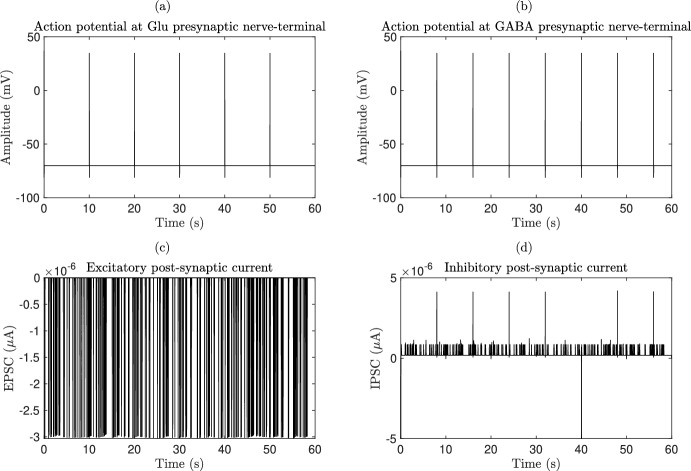
Neuronal spiking activity and synaptic currents for a representative quadripartite synapse system. **(a)** Action potential at the glutamatergic presynaptic nerve terminal, **(b)** Action potential at the GABA presynaptic nerve terminal, **(c)** EPSC, and **(d)** IPSC

**Fig. 10:**
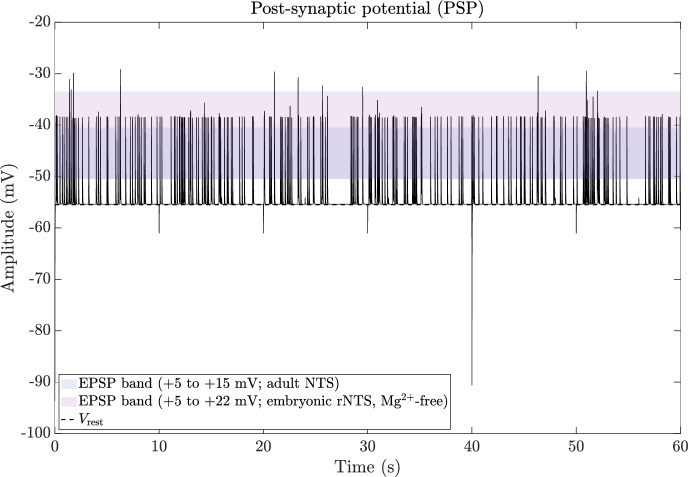
PSP for a representative quadripartite synapse system with overlaid experimental reference bands. The blue band (+5 to +15 mV above $V_{\textrm{rest}}$) represents typical tract-evoked EPSP amplitudes reported for adult NTS neurons under baseline physiological conditions (Zhou et al. 1997). The purple band (+5 to +22 mV) reflects larger EPSPs observed in embryonic rostral NTS when NMDA contributions are revealed in $\hbox {Mg}^{2+}$-free ACSF (suppressed again by APV) (Suwabe et al. 2013)

The neuronal spiking activity associated with a representative quadripartite synapse system is shown in Figures 9 and 10. These figures provide an in-depth view of the electrical dynamics and synaptic currents that govern the interaction between excitatory and inhibitory components in the synapse.

Figure 9 illustrates the temporal evolution of the AP and synaptic currents. Panel (a) depicts the AP at the Glu presynaptic nerve terminal, characterized by rapid depolarization and repolarization cycles. This reflects the excitation dynamics triggered by Glu neurotransmitter release, essential for synaptic activation and downstream signaling. Panel (b) shows the AP at the GABA presynaptic nerve terminal, where the amplitude and timing differ from those of the Glu terminal, highlighting the distinct role of inhibitory neurotransmission in modulating the overall synaptic response.

Panel (c) presents the excitatory post-synaptic current (EPSC), which results from the cumulative release of Glu neurotransmitters into the synaptic cleft and their binding to post-synaptic receptors. The EPSC curve demonstrates the temporal summation of excitatory inputs, driving the post-synaptic neuron toward depolarization. In contrast, panel (d) displays the inhibitory post-synaptic current (IPSC), generated by the release of GABA neurotransmitters. The IPSC curve represents the inhibitory effect on the post-synaptic neuron, counteracting excitatory signals and maintaining neuronal balance.

Figure 10 focuses on the post-synaptic potential (PSP), which integrates the effects of excitatory and inhibitory inputs at the post-synaptic neuron. The PSP curve exhibits the combined depolarization and hyperpolarization phases, illustrating how synaptic inputs modulate the membrane potential and influence the likelihood of AP generation. For reference, the shaded bands overlay experimentally reported excitatory post-synaptic potential (EPSP) amplitudes: a 5–15 mV range for tract-evoked responses in adult NTS neurons under baseline conditions (Zhou et al. 1997), and a wider 5–22 mV envelope observed in embryonic rostral NTS when NMDA contributions are revealed in Mg$^{2+}$-free artificial cerebrospinal fluid (ACSF) and suppressed again by (2R)-amino-5-phosphonopentanoate (APV) (Suwabe et al. 2013). The simulated PSP excursions lie within the adult NTS reference band, while the embryonic NMDA-unblocked band is included as an upper benchmark for comparison.

### Astrocytic calcium and gliotransmitter signaling

**Fig. 11:**
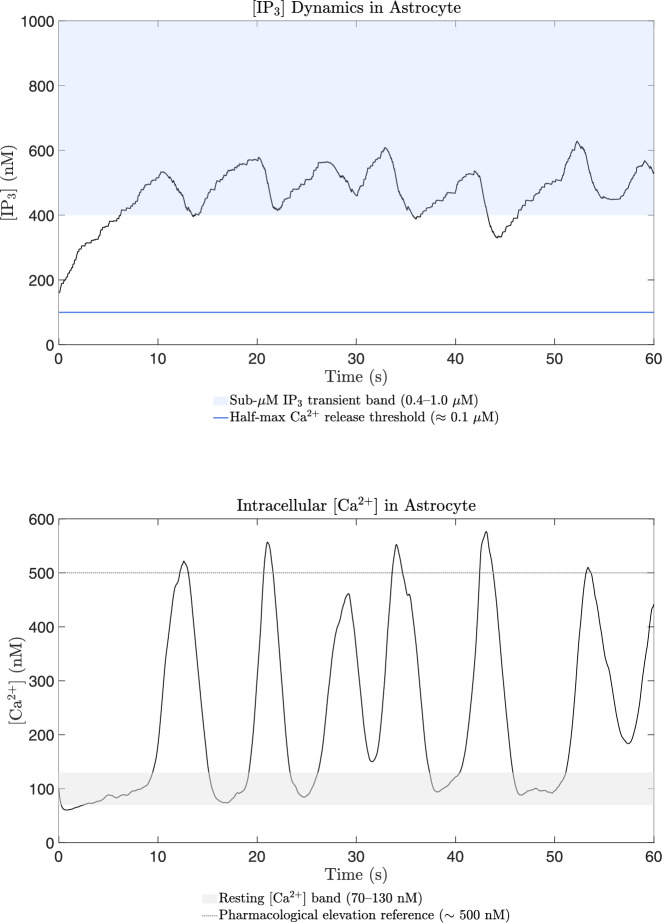
Astrocytic signaling dynamics with experimental reference bands. Top: $[\textrm{IP}_3]$ dynamics overlaid with a sub-micromolar transient band (0.4–1.0 $\mu$M) and a horizontal reference indicating the half-maximal threshold for IP$_3$-evoked Ca$^{2+}$ release ($\textrm{EC}_{50}\approx 0.1~\mu \textrm{M}$) (Fink et al. 1999; Montes et al. 2018). Bottom: intracellular astrocytic $[\textrm{Ca}^{2+}]$ with a resting band of 70–130 nM consistent with experimental measurements and reviews (Shigetomi et al. 2016; Bambrick et al. 1997; Parpura and Haydon 2000); a dotted line at $\sim$500 nM provides an optional pharmacological reference under SERCA inhibition

Astrocytes serve as pivotal mediators within the NGVU, responding to neuronal activity with coordinated $\hbox {IP}_3$–$\hbox {Ca}^{2+}$ signaling cascades that regulate gliotransmitter release and influence vascular responses. Figure 11 illustrates the temporal evolution of astrocytic [$\hbox {IP}_3$] and [$\hbox {Ca}^{2+}$] concentrations with experimental reference bands. The top panel shows [$\hbox {IP}_3$] dynamics overlaid with a sub-micromolar transient band (0.4–1.0 $\mu \hbox {M}$) and a horizontal reference at the half-maximal threshold for $\hbox {IP}_3$-evoked $\hbox {Ca}^{2+}$ release ($\text {EC}_{50}\approx 0.1~\mu \hbox {M}$), providing external benchmarks for model calibration and validation (Fink et al. 1999; Montes et al. 2018). The bottom panel displays intracellular [$\hbox {Ca}^{2+}$] with a resting band of 70–130 nM derived from experimental measurements and reviews, and an optional pharmacological reference near 500 nM under SERCA inhibition (Shigetomi et al. 2016; Bambrick et al. 1997; Parpura and Haydon 2000). The observed $\hbox {Ca}^{2+}$ transients exhibit the expected rapid rise and slower decay, reflecting $\hbox {IP}_3$-mediated release from internal stores followed by sequestration and extrusion. These constraints place our simulated trajectories within physiologically plausible regimes, supporting the role of astrocytes as modulators of neuron–glia–vascular communication through gliotransmission and metabolic/vascular regulation.

### Neurovascular response

**Fig. 12:**
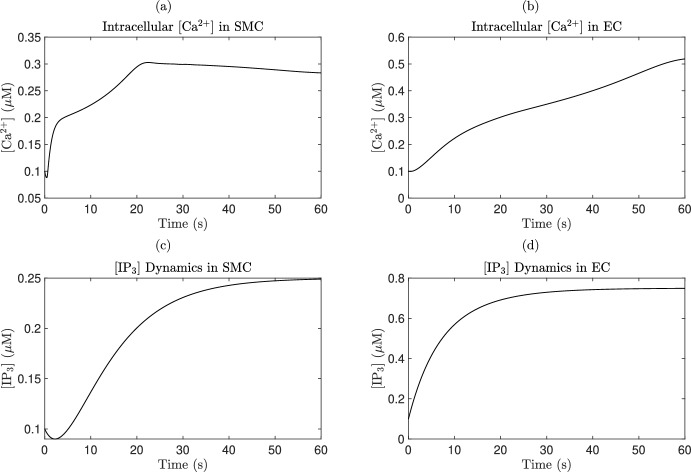
Calcium and $\hbox {IP}_3$ dynamics in vascular cells: **(a)** and **(b)** show intracellular calcium concentration in SMCs and endothelial cells, alongside **(c)** and **(d)** $\hbox {IP}_3$ signaling dynamics in these cells, consistent with neurovascular coupling models (Dormanns et al. 2015, 2016)

Figure 12 illustrates the temporal dynamics of calcium and $\hbox {IP}_3$ signaling within vascular SMCs and ECs, key components in the neurovascular coupling process. The intracellular calcium concentration in SMCs exhibits oscillatory behavior over time, which is critical for regulating vascular tone. These calcium dynamics mediate vasoconstriction and vasodilation by modulating the contractile state of SMCs, thereby directly influencing the radius of the blood vessel. Similarly, the intracellular calcium signals in ECs play a complementary role by driving the release of vasoactive mediators such as NO and prostacyclin. These mediators propagate the effects of endothelial activation to adjacent SMCs, ensuring coordinated vascular responses.

The $\hbox {IP}_3$ signaling pathways, shown in the same figure, highlight the molecular mechanisms underlying calcium mobilization. In SMCs, $\hbox {IP}_3$ variations reflect the activation of G-protein-coupled receptors and subsequent release of calcium from intracellular stores, such as the sarcoplasmic reticulum. In ECs, $\hbox {IP}_3$ signaling serves as a critical intermediary, linking upstream signaling events to calcium mobilization and facilitating the generation of calcium waves along the endothelium, in line with computational models of neurovascular coupling (Dormanns et al. 2015, 2016).

**Fig. 13:**
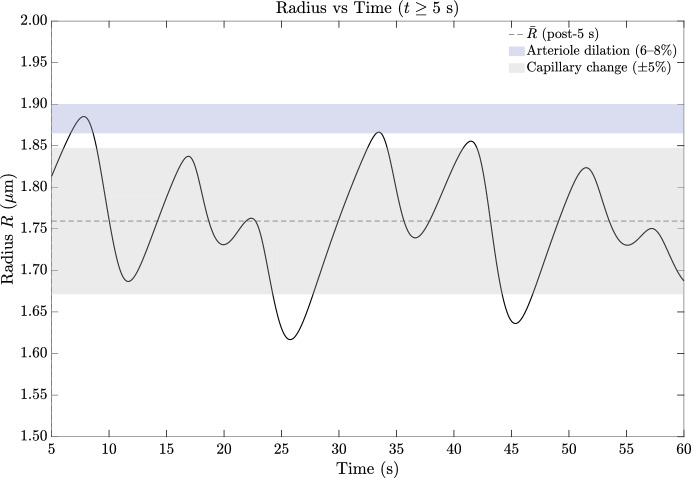
Temporal vessel radius with experimentally grounded reference bands. The blue band (6–8%) illustrates a representative arteriolar dilation during normal sensory activation, consistent with two-photon/NVU studies reporting small–to–moderate ($\lesssim$10%) arteriolar dilations driven by neuronal/astrocytic signalling (Cai et al. 2018; Filosa et al. 2006). The grey band ($\pm 5\%$) denotes a conservative physiological envelope for capillaries, reflecting that capillary calibre changes during neurovascular coupling are modest while most diameter change appears in upstream arterioles via conducted responses (Longden et al. 2017; Rungta et al. 2018; Hall et al. 2014)

Figure 13 presents the dynamic changes in blood vessel radius as a result of neurovascular coupling. The variations in vessel radius reflect the synergistic effects of calcium and inositol trisphosphate ($\hbox {IP}_3$) signaling on vascular tone regulation. Vasodilation and vasoconstriction are observed in response to these intracellular signals, demonstrating the capacity of the vascular network to adapt to changes in neuronal and glial activity. This response ensures efficient oxygen and nutrient delivery to tissues, particularly in metabolically active regions. Because the absolute initial radius is uncertain, we discard an initial settling period of 5 s, compute the mean radius ${\bar{R}}$ over the post–5 s interval, and report and visualize dynamics with respect to this baseline. The plot overlays experimentally grounded bands: a 6–8% arteriolar dilation band and a conservative $\pm 5\%$ capillary envelope. As the simulated trace corresponds to a capillary segment receiving arterial inflow, comparison should primarily be made to the capillary band; the arteriole band is provided as an upstream benchmark.

The results depicted in these figures validate the integrated neurovascular model by reproducing key physiological behaviors observed in experimental studies. The coupling between neuronal activation, astrocytic signaling, and vascular responses provides a comprehensive framework for understanding neurovascular coupling mechanisms and their implications in health and disease. The agreement of the simulated capillary dynamics with the conservative capillary envelope, together with arteriole benchmarks, provides an additional consistency check for physiological plausibility.

### Parameter study and sensitivity of post-transient radius dynamics

To quantify how uncertainty in wall–mechanics parameters propagates to the vessel response driven by the NGVU, we performed a combined local and global sensitivity analysis focused on the time-varying radius $R(t)$ after the initial transient. The response metric is the amplitude of the post-transient oscillation around its own mean: letting $T_0=5~\textrm{s}$ and $t_{\textrm{end}}=60~\textrm{s}$, the time-weighted mean $\bar{R}$ is

$$ {{\bar{R}}} \;=\; \frac{1}{t_{\textrm{end}}-T_0}\int _{T_0}^{t_{\textrm{end}}} R(t)\,\textrm{d}t, $$

and the objective is

$$ A \;=\; \max _{t\in [T_0,\,t_{\textrm{end}}]} \bigl |R(t)-{{\bar{R}}}\bigr |. $$

**Fig. 14:**
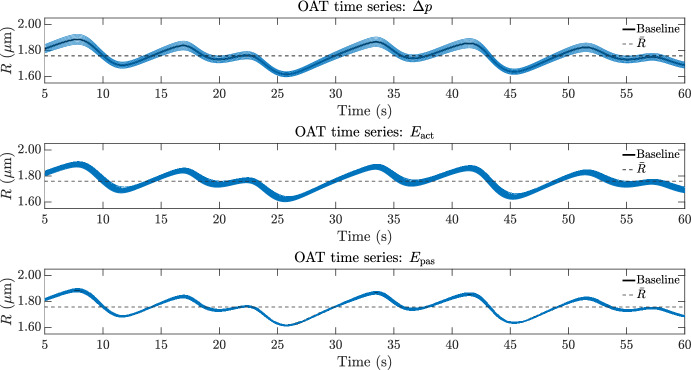
OAT time-series sensitivity of radius $R(t)$ for $\pm 30\%$ multiplicative scaling of a single parameter at a time. Baseline trace and post-$5~\textrm{s}$ mean are overlaid; only $t\ge 5~\textrm{s}$ is plotted. Rows correspond to $\Delta p$, $E_{\textrm{act}}$, and $E_{\textrm{pas}}$, respectively

Unless stated otherwise, results are reported as a relative change with respect to the baseline amplitude $A_0$: $\Delta A \equiv 100\,\bigl (A/A_0-1\bigr )\,[\%]$. This definition suppresses early transients and isolates the magnitude of the neurovascularly driven modulation of $R(t)$, which is the quantity of direct interest in our model–data comparisons. Note that $A$ is computed about each run’s own post-transient mean; therefore, parameters that primarily translate the mean of $R(t)$ (most notably $E_{\textrm{act}}$) can produce wide raw time-series bands in Figure 14 without implying a larger $\Delta A$, whereas parameters that change the excursion size about the mean (e.g. $E_{\textrm{pas}}$) directly affect $\Delta A$.

**Fig. 15:**
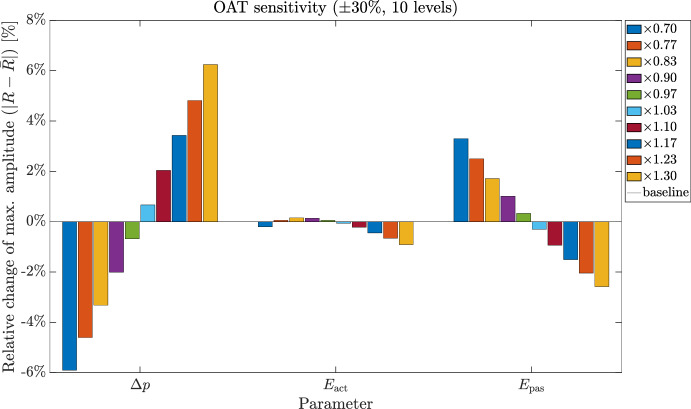
Grouped OAT response for the objective $\Delta A$ (relative change of post-$5~\textrm{s}$ amplitude) across ten factors in $[0.7,1.3]$ for each parameter ($\Delta p$, $E_{\textrm{act}}$, $E_{\textrm{pas}}$). Increasing $\Delta p$ enlarges the amplitude, whereas increasing either modulus reduces it; the effect of $E_{\textrm{act}}$ exceeds that of $E_{\textrm{pas}}$

**Fig. 16:**
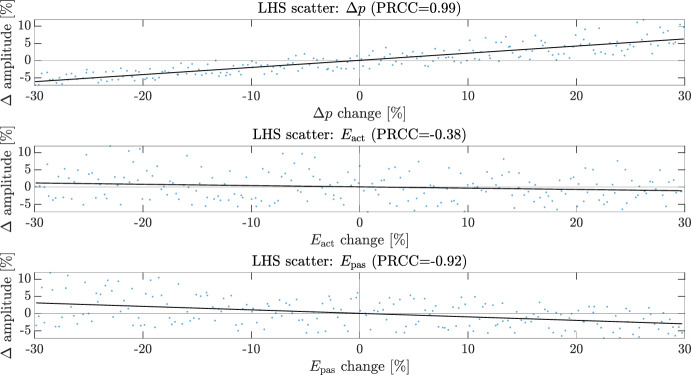
Global uncertainty analysis via LHS ($N=200$): marginal scatter of parameter percent change versus $\Delta A$ for $\pm 30\%$ ranges around baseline. Linear trends are evident: positive for $\Delta p$ and negative for $E_{\textrm{act}}$ and $E_{\textrm{pas}}$, with limited curvature or interaction signatures

**Fig. 17:**
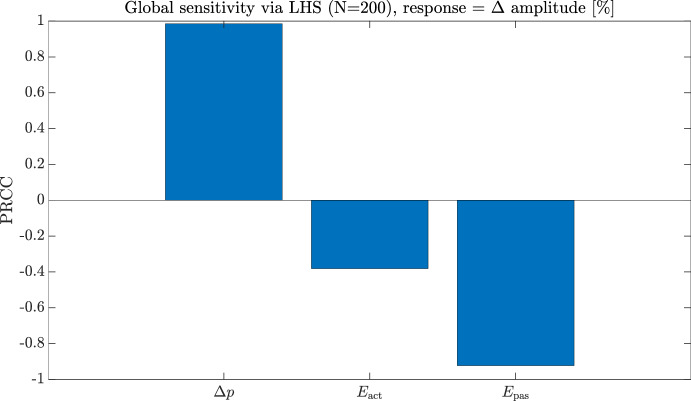
PRCC summary ($N=200$ LHS samples) for the response $\Delta A$. Ordering and signs corroborate the OAT analysis: $\Delta p$ dominates positively, followed by negative contributions from $E_{\textrm{act}}$ and $E_{\textrm{pas}}$

**Fig. 18:**
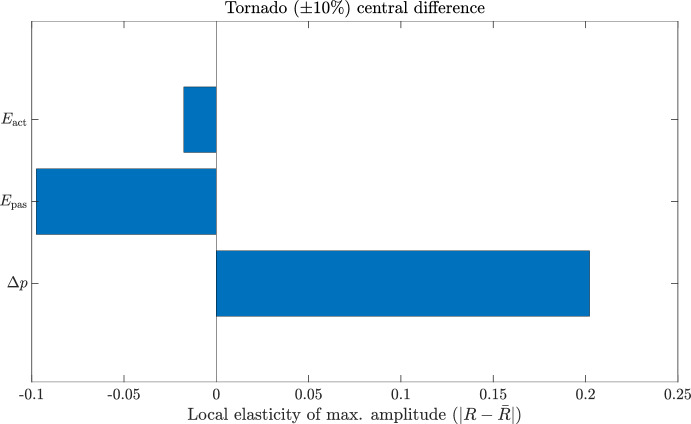
Local elasticities (tornado) from symmetric $\pm 10\%$ perturbations at baseline for $\Delta p$, $E_{\textrm{act}}$, and $E_{\textrm{pas}}$. The elasticity of $\Delta p$ is largest and positive; both moduli are negative, with greater magnitude for $E_{\textrm{act}}$

We probed three scalar parameters from the Kelvin–Voigt wall law that are both mechanically interpretable and practically identifiable in our setting: an effective transmural pressure scale $\Delta p$, the active elastic modulus $E_{\textrm{act}}$, and the passive elastic modulus $E_{\textrm{pas}}$. The viscoelastic damping coefficient was held fixed to avoid confounding stiffness–damping trade-offs. For each parameter set, the coupled NGVU ODEs were integrated over $[0,60]~\textrm{s}$ with identical solver tolerances to the baseline, and $R(t)$ was post-processed precisely as above. We used two complementary designs. First, a one-at-a-time (OAT) sweep, in which a single parameter was scaled by ten equally spaced factors over $[0.7,\,1.3]$ while the others were kept at baseline, and the full time series as well as $\Delta A$ were recorded (Figures 14, 15). The apparent broader spread under $E_{\textrm{act}}$ in Figure 14 is explained by a shift of the post-transient mean relative to the baseline overlay; because our objective re-centers each run on its own mean, this mean shift does not translate into a larger $\Delta A$, which remains more sensitive to $E_{\textrm{pas}}$ than to $E_{\textrm{act}}$ in the explored range. Second, a global design based on Latin hypercube sampling (LHS) with $N=200$ samples drawn independently and uniformly from $\pm 30\%$ hyperrectangles around the baseline values; we summarized main effects with rank-based partial correlations (PRCCs) and visualized response structure with marginal scatter plots and a PRCC bar chart (Figures 16, 17). In addition, we computed local elasticities by a symmetric $\pm 10\%$ central difference at baseline,

$$ S \;=\; \frac{p_0}{A_0}\;\frac{A^{(+)}-A^{(-)}}{p_0\,(f_+ - f_-)}, $$

where $p_0$ is the baseline parameter value and $f_\pm \in \{0.9,1.1\}$ are the two scale factors (Figure 18). Together, these three views (OAT, PRCC, tornado) give shape, rank and local slope information, respectively.

The OAT time-series panels show that scaling $\Delta p$ primarily modulates the excursion of $R(t)$ about its mean without substantially altering the qualitative waveform, whereas scaling the moduli compresses or releases that excursion by stiffening or softening the wall (Figure 14). Correspondingly, the grouped OAT bars for $\Delta A$ are nearly monotone in each factor: increasing $\Delta p$ increases amplitude, while increasing either $E_{\textrm{act}}$ or $E_{\textrm{pas}}$ decreases it (Figure 15). The effect of $E_{\textrm{act}}$ is visibly larger than that of $E_{\textrm{pas}}$, consistent with the active branch setting the effective compliance in the physiologically relevant operating range of $R$.

The global LHS analysis corroborates these trends and controls for parameter co-variation. The marginal scatter plots display clear, approximately linear relationships between the percent change in each parameter and $\Delta A$, with a positive slope for $\Delta p$ and negative slopes for both moduli (Figure 16). The PRCC summary ranks $\Delta p$ as the dominant contributor to $\Delta A$, followed by $E_{\textrm{act}}$ and then $E_{\textrm{pas}}$ (Figure 17). Importantly, the absence of pronounced curvature or funneling in the scatter panels suggests limited nonlinearity and weak interaction effects within the explored $\pm 30\%$ neighborhood, lending confidence to the PRCC interpretation. Finally, the tornado diagram of local elasticities reproduces the same ordering at baseline, with $\Delta p$ exerting the largest positive elasticity and both elastic moduli exerting negative elasticities, the magnitude being larger for $E_{\textrm{act}}$ (Figure 18). This alignment across local (tornado), semi-local (OAT), and global (LHS/PRCC) diagnostics indicates that, in our NGVU-driven dynamics, the amplitude of post-transient radius modulation is primarily pressure-driven and secondarily stiffness-limited by the active mechanics of the wall.

From an interpretive standpoint, these findings have two practical consequences for model calibration and uncertainty reduction. First, the dominant influence of $\Delta p$ on $\Delta A$ implies that experimental constraints on local transmural pressure (or equivalently, on the effective pressure drop entering the microvascular segment) will directly sharpen predictions of functional radius modulation. Second, separating the roles of $E_{\textrm{act}}$ and $E_{\textrm{pas}}$ suggests that dynamic dilation amplitudes are more informative about active tone than about purely passive tissue properties; independent measurements of passive modulus would therefore be especially valuable for breaking residual stiffness identifiability. In summary, the parameter study reveals a mechanically coherent picture: pressure sets the drive, active stiffness sets the gain, and passive stiffness provides a smaller, secondary brake–an organization that is consistent with the qualitative behavior seen in the OAT time series, the monotone OAT response curves, the LHS/PRCC summaries, and the tornado ranking (Figures 14–18).

### Microcirculation on a representative unit of the DVC

Our computations extend the geometry of the *brain99* dataset to simulate the electromechanical coupling between neuro-glial activity and vascularization within the DVC. This model specifically captures the interaction between astrocytic and neuronal activity and the resulting dynamic changes in blood vessel radii. Blood flow dynamics and oxygen transport are analyzed within the vascular network to assess the influence of neuro-glial signals on vascular responses. The results demonstrate how capillary networks adapt to facilitate metabolic activity and neuronal function, while the hierarchical vascular structure ensures efficient blood supply and flow redistribution. This study particularly highlights the integration of macro- and microcirculation models to validate the mechanistic framework for neuro-glial-vascular coupling in the DVC.

**Fig. 19:**
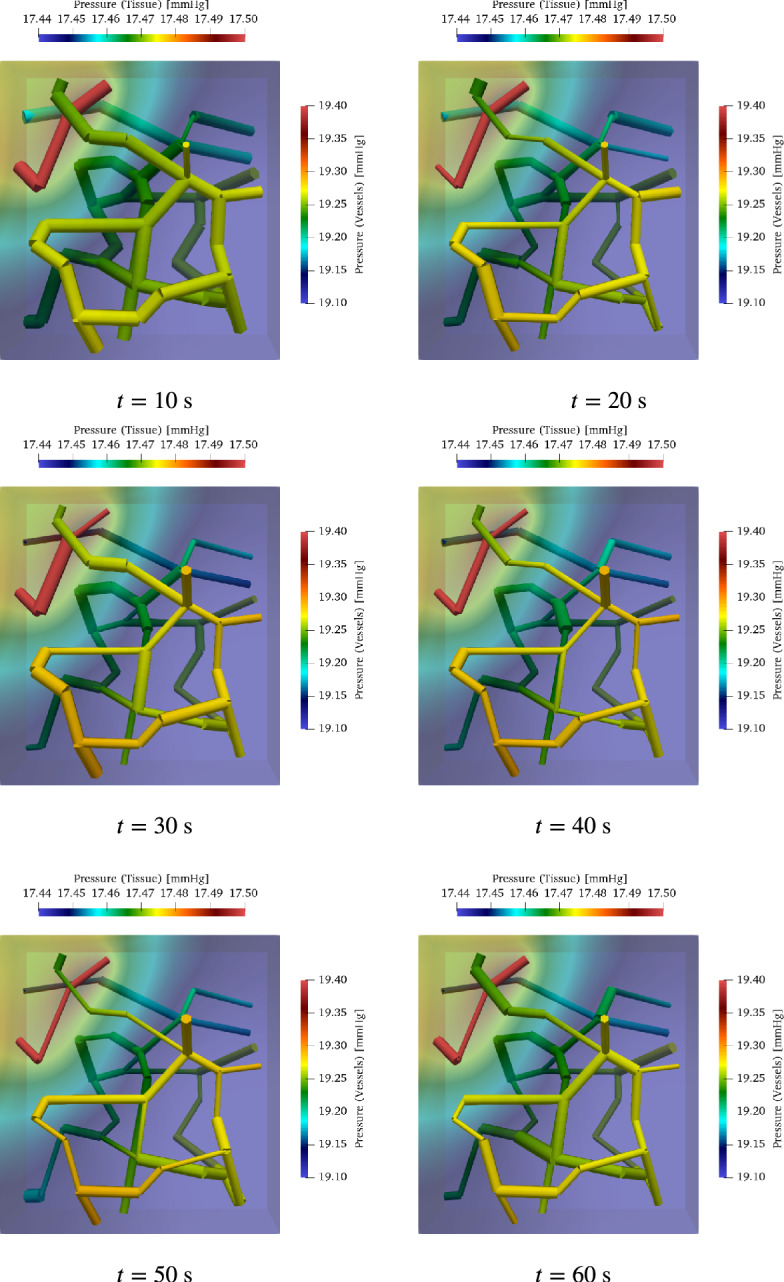
Time-resolved simulation results of blood flow within the DVC microcirculation network. A cross-sectional view is obtained by slicing the computational unit (a unit cube) along its central horizontal plane. The color map represents the local blood flow velocity magnitude, highlighting how neuro–glial–vascular coupling causes dynamic redistribution of flow via vessel dilation and constriction in both capillaries and larger vessels

**Fig. 20:**
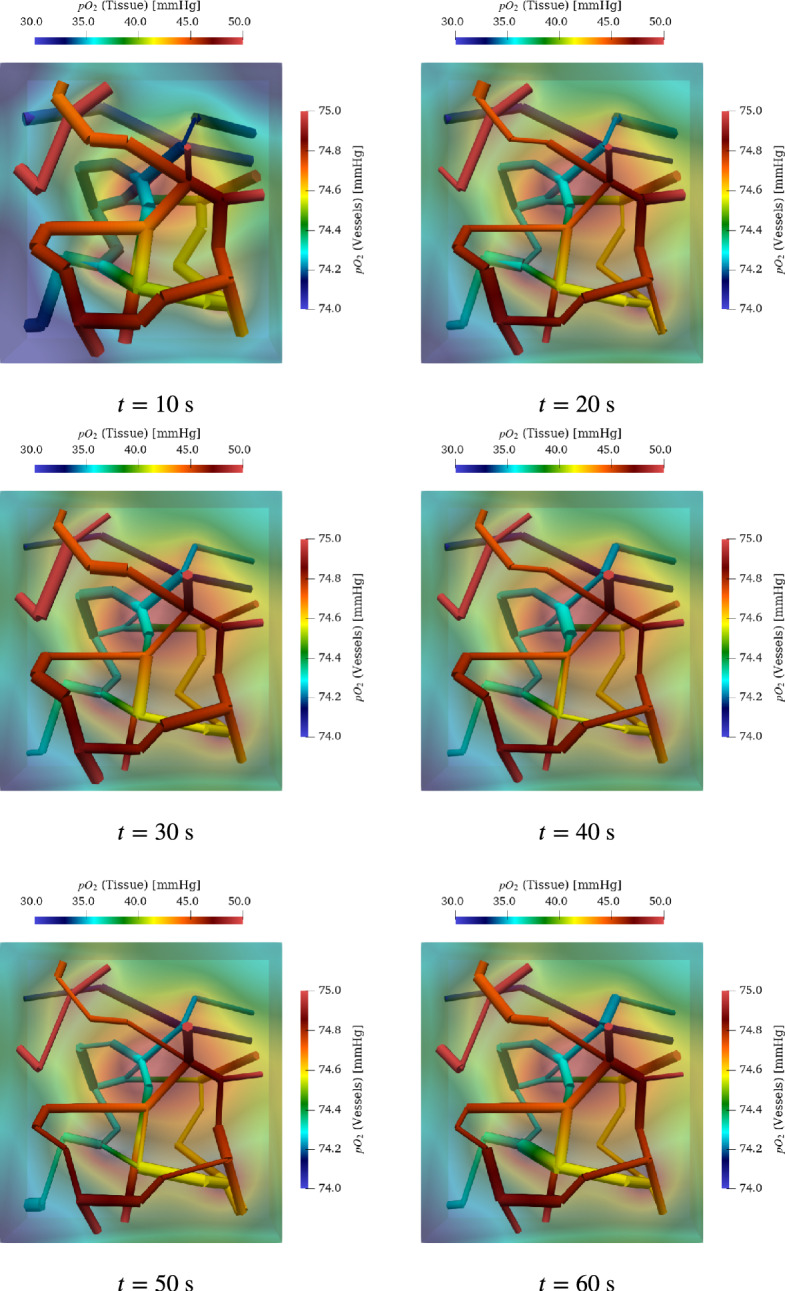
Time-resolved simulation results of oxygen distribution within the DVC microcirculation network. A cross-sectional slice through the center of the computational domain (unit cube) is rendered in 3D, with colors indicating the oxygen partial pressure ($pO_2$) in blood vessels and the surrounding tissue. This view captures the dynamics of oxygen delivery and diffusion via the 1D–3D coupling model, illustrating the impact of vessel diameter changes driven by neuro–glial interactions

The figures below illustrate the temporal evolution of blood flow patterns (Figure 19) and oxygen distribution (Figure 20) within the DVC microvascular network. These results reveal spatiotemporal variations in hemodynamics and oxygen delivery, computed based on the coupled neuro-glial-vascular framework. Figure 19 highlights how neuro-glial activity drives dynamic changes in blood flow, with vessel dilation and constriction contributing to flow redistribution across the network. Similarly, Figure 20 visualizes oxygen transport and diffusion within the vascular network and surrounding tissue, emphasizing the role of capillaries in ensuring adequate oxygenation. Together, these simulations provide detailed insights into the dynamic interplay between neuronal activation, astrocytic metabolism, and vascular responses within the DVC.

## Discussion

The numerical results presented in this work illustrate how the integration of neuronal activity, astrocytic signaling, and vascular dynamics can yield a comprehensive understanding of neuro-glial-vascular interactions in the DVC. By explicitly simulating neurotransmission, neuron–astrocyte $\hbox {Ca}^{2+}$ signaling, and vessel tone regulation, the model captures key physiological features of functional hyperemia, highlighting the system’s ability to meet local metabolic demands. Coupling synaptic events, astrocytic gliotransmission, and vascular responses underscores the regulatory feedback loops that govern cerebral blood flow in health and disease. Relative to the conceptual, network-wide NGVU framework of Kugler et al. (Kugler et al. 2021), our contribution operationalizes a DVC-specific, dynamical chain that links synaptic activity to vessel mechanics via an integrated quadripartite synapse and multiscale flow, yielding quantitative, testable predictions for stimulus–radius coupling in brainstem microcirculation.

In contrast to stimulus-to-hemodynamic balloon and Windkessel formulations that lump vascular compliance and ignore explicit cell-to-vessel pathways, the present 3D–1D–0D framework resolves the chain from synaptic drive through astrocytic $\hbox {IP}_3$–$\hbox {Ca}^{2+}$ signaling to vessel wall mechanics on a realistic microvascular graph. Compared with endothelial and pericyte signaling models formulated on idealized segments, our implementation computes spatially distributed radius and flow fields while retaining mechanistic links to neurotransmission and gliotransmission. Relative to reconstruction and workflow ecosystems that emphasize large-scale assembly and interoperability, our contribution is a NGVU-specific coupling pattern tailored to the DVC that can be embedded in such pipelines to generate testable predictions at microvascular resolution.

Despite these promising findings, certain limitations must be acknowledged. The current study relies on simplified geometries and assumptions for macrocirculation, and we have primarily focused on the microcirculatory block derived from the *brain99* dataset. This choice improves tractability, but future models should incorporate more detailed anatomical reconstructions and account for heterogeneities at multiple scales (e.g., morphological variations in larger vessels or region-specific neuronal phenotypes). Additionally, improving the fidelity of cell-specific kinetics and including metabolic constraints–such as oxygen and glucose availability–could further enhance the model’s predictive power. To quantify robustness toward that translational goal, we added a parameter sensitivity and uncertainty analysis of post-transient radius dynamics, clarifying which wall-mechanics parameters most control amplitude and where experimental constraints would most reduce prediction spread (*cf.* Section 4.6). As future work, we aim to move toward human validation using appropriate datasets, and to drive the NGVU in the DVC with brain-gut axis inputs, including vagal afferent signals induced by gastric wall deformation and endocrine cues such as ghrelin and leptin, to identify operating regimes that link neuronal activity, astrocytic $\hbox {Ca}^{2+}$ dynamics, vessel tone, perfusion, and metabolic demand.

Beyond these limitations, the NGVU formalism is designed to generalize across species and brain disorders by preserving structure while retuning biophysical priors such as vessel wall mechanics, receptor expression profiles, baseline tone, and boundary conditions. On the cohort side, harmonized MRI pipelines spanning disorders and the lifespan provide consistent macro- and microvascular readouts–perfusion, vessel-calibre proxies, and tissue-oxygenation surrogates–that map naturally to our model observables for calibration and validation (Koike et al. 2021). On the perturbation side, GABAergic signaling within the microbiome–gut–brain axis modulates central circuits and offers concrete NGVU-relevant inputs via vagal afferents in the DVC, enabling tests of model transfer and sensitivity in health and disease (Belelli et al. 2025). Together these directions outline a practical path to species translation from rat to human and to cross-disorder applications by combining harmonized phenotypes with pathway-specific perturbations within the same mechanistic scaffold.

## Conclusion

In conclusion, we have developed a modular, multi-scale modeling framework that unifies neuronal spiking, astrocytic calcium dynamics, and vascular tone regulation within the dorsal vagal complex. Our work introduces a coupled multi-scale model that provides a seamless transition from systemic hemodynamics to local capillary dynamics. In this framework, models for flow and transport of different dimensions are coupled, resulting in a 3D-1D-0D system. By embedding a quadripartite synapse model and linking it to a macro-/microcirculatory description, we demonstrate that neuro-glial-vascular coupling emerges through interactive feedback processes. The proposed approach provides a valuable platform for studying neurovascular regulation, investigating pathological disruptions of neurovascular homeostasis, and designing future experiments. Looking ahead, the framework could be linked to standardized cohort-level imaging readouts and to controlled physiological perturbations to assess transfer across species and diagnoses. Importantly, this work provides an integrated model for the DVC, quantitatively capturing the interplay among neural, glial, and vascular components. Our simulation results reproduce physiologically realistic hemodynamic and synaptic behaviors in accordance with established textbook data, providing an initial validation of the model. We envisage that further refinement and extension of this model, for example by including patient-specific characteristics such as receptor heterogeneity, will offer deeper insights into cerebrovascular regulation and guide novel therapeutic strategies for various neurological conditions. Moreover, our simulation results establish a robust computational foundation for future translational investigations into both normal and pathological brain function. Finally, the generality of our methodology indicates that a similar modeling framework can be applied to other cortical areas, further broadening the scope of neurovascular research.

## Data Availability

The capillary network used in this work (*brain99*) is publicly available from the University of Arizona repository: https://sites.arizona.edu/secomb/3d-network-data-brain-1999/. The simulation code and workflow for the NGVU experiments are available at: https://github.com/alhermann/ngvu-multiscale-3d1d0d.
